# Targeting APE1 endonuclease activity impairs metastasis and enhances genotoxic therapy response in pancreatic cancer

**DOI:** 10.1186/s13046-026-03705-7

**Published:** 2026-04-02

**Authors:** Eyram K. Kpenu, Mahmut Mijit, Silpa Gampala, Sheng Liu, Jun Wan, Randall S. Wireman, Jacqueline Peil, Dana K. Mitchell, Sanya Haiaty, Rajesh Sardar, Akanksha Sharma, Millie M. Georgiadis, Melissa L. Fishel, Mark R. Kelley

**Affiliations:** 1 Department of Biochemistry, Molecular Biology and Pharmacology, Indiana University School of Medicine, Indianapolis, IN USA; 2 Department of Pediatrics and Herman B Wells Center for Pediatric Research, Indiana University School of Medicine, Indianapolis, IN USA; 3 Simon Comprehensive Cancer Center, Indiana University School of Medicine, Indianapolis, IN USA; 4 Department of Medical and Molecular Genetics, Indiana University School of Medicine, Indianapolis, IN USA; 5 Department of Chemistry and Chemical Biology, Indiana University- Indianapolis, Indianapolis, IN USA

**Keywords:** Pancreatic ductal adenocarcinoma (PDAC), Apurinic/Apyrimidinic Endonuclease 1 (APE1), Base excision repair (BER), Endonuclease activity, Metastasis, Temozolomide (TMZ), Mitochondrial DNA damage, Genotoxic stress

## Abstract

**Background:**

Pancreatic ductal adenocarcinoma (PDAC) is a highly deadly cancer with limited treatment options. The base excision repair (BER) pathway, crucial for fixing DNA abasic sites, is driven by apurinic/apyrimidinic endonuclease 1 (APE1). While APE1 redox function in PDAC has been extensively studied, its endonuclease activity in PDAC homeostasis and therapeutic response remains poorly understood. We created stable, homozygous APE1 endonuclease-reduced PDAC cell lines to examine the effects of impaired BER activity on pancreatic cancer growth and response to treatment.

**Methods:**

CRISPR/Cas9-mediated editing was used to introduce an E96A mutation into the Pa03C PDAC cell line, generating three clonal mutant cell lines: E96A B1, E96A B4, E96A G8. APE1 expression and activity were verified in vitro through biochemical assays. Cellular responses to genotoxic stress were examined using cytotoxicity, colony formation, and DNA damage assays. Transcriptomic changes were evaluated via RNA sequencing. In vivo tumor growth and metastatic dissemination were studied in orthotopic PDAC mouse models, with and without treatment.

**Results:**

The E96A mutant cell lines exhibited significantly decreased endonuclease activity but showed no changes in redox signaling or APE1 protein expression. Short-term cytotoxic assays revealed no enhancement in acute sensitivity; however, long-term assessment demonstrated a proliferative defect and a vulnerability to genotoxic stress. Quantitation of nuclear and mitochondrial DNA damage showed mutant cells accumulated significantly more damage to both genomes compared to controls. Transcriptomic analysis revealed that the mutant cell lines maintain a stressed phenotype at baseline, which becomes more pronounced following DNA damage. In vivo, E96A mutants had notably lower tumor burden and metastasis without treatment, and the mutation potentiated the effect of the alkylating drug temozolomide, which inhibited tumor growth and metastasis in a dose-dependent manner.

**Conclusion:**

We established the first stable human PDAC cell models deficient in APE1 endonuclease activity. Our findings demonstrate that selective impairment of APE1’s DNA repair function expands therapeutic options by lowering the threshold for effective processing of DNA damage, validating combination treatments with targeted inhibitors and DNA-damaging agents. Targeting APE1 endonuclease activity represents a promising therapeutic strategy for PDAC, capable of suppressing metastatic spread and enhancing tumor responsiveness to genotoxic therapies.

**Supplementary Information:**

The online version contains supplementary material available at 10.1186/s13046-026-03705-7.

## Background

Pancreatic ductal adenocarcinoma (PDAC) is the most common type of pancreatic cancer, representing over 90% of all cases [1]. It is a clinically challenging malignancy with an increasing incidence and is projected to become the second leading cause of cancer-related deaths by 2030 [2, 3]. PDAC’s low 5-year survival rate of 13% underscores the urgent need to explore new treatment options. Given that it exists in a harsh tumor microenvironment (TME) consisting of inflammation, hypoxia, and high levels of reactive oxygen species (ROS), all conditions that exacerbate genotoxic stress, PDAC may be vulnerable to strategies that target inherent weaknesses in its DNA repair mechanisms [4].

Cellular DNA repair comprises a complex network of pathways and repair factors that mirrors the diverse range of DNA damage. In cancer, increased DNA damage often leads to the upregulation of repair mechanisms, making these pathways mainstay targets for therapy [5, 6]. The current first-line treatment for PDAC, FOLFIRINOX, is a combination regimen that includes multiple genotoxic agents that induce a broad spectrum of DNA damage [5, 7, 8]. The base excision repair pathway (BER) is the main mechanism for fixing damaged bases and abasic (AP) sites [9, 10]. In a normal cell, about 10,000 to 20,000 AP sites are produced daily, either through spontaneous base loss or, more frequently, through lesioned base removal by DNA glycosylases, making BER critical for normal cell homeostasis [11, 12]. The increased levels of genotoxic stress in PDAC lead to an even greater accumulation of lesioned bases, especially oxidized base damage, increasing reliance on BER and making it a promising target for PDAC therapies [13, 14].

The rate-limiting process in BER is facilitated by Apurinic/apyrimidinic endonuclease 1/Redox effector 1 (APE1/Ref-1), hereafter APE1 [15, 16]. While it can also function as a redox signaling protein, APE1 is the primary endonuclease in the canonical BER pathway that recognizes AP sites and cleaves the DNA phosphate backbone adjacent to them, enabling downstream gap filling and strand ligation [17]. A conserved pathway, BER is essential for cell survival, and complete APE1 removal is lethal in most contexts [12, 18].

APE1 is often overexpressed in cancers, with both redox and endonuclease functions (which operate independently) exploited by oncogenic cells for survival [19–21]. By leveraging APE1’s redox function, tumor cells can alter transcriptional and metabolic pathways to drive growth [22–24]. A previous study using APE1^C65A^ redox-deficient mutants demonstrated that selective impairment of APE1’s redox function reduces tumor growth and metastasis in PDAC models in vivo [25], establishing the precedent for studying APE1’s dual functions independently.

Yet, while the network of APE1’s redox function in PDAC homeostasis is becoming better understood, the contribution of its endonuclease activity towards PDAC tumor homeostasis remains comparatively less clear and has not been studied in vivo using stable genetic models. In line with this, pharmacologic efforts to target APE1 endonuclease activity have lagged behind redox-directed strategies [24]. Given its unique role in safeguarding genomic integrity under oxidative stress, targeting APE1 to inhibit BER represents a promising, yet underexplored, therapeutic opportunity in PDAC [21, 26].

**Fig. 1:**
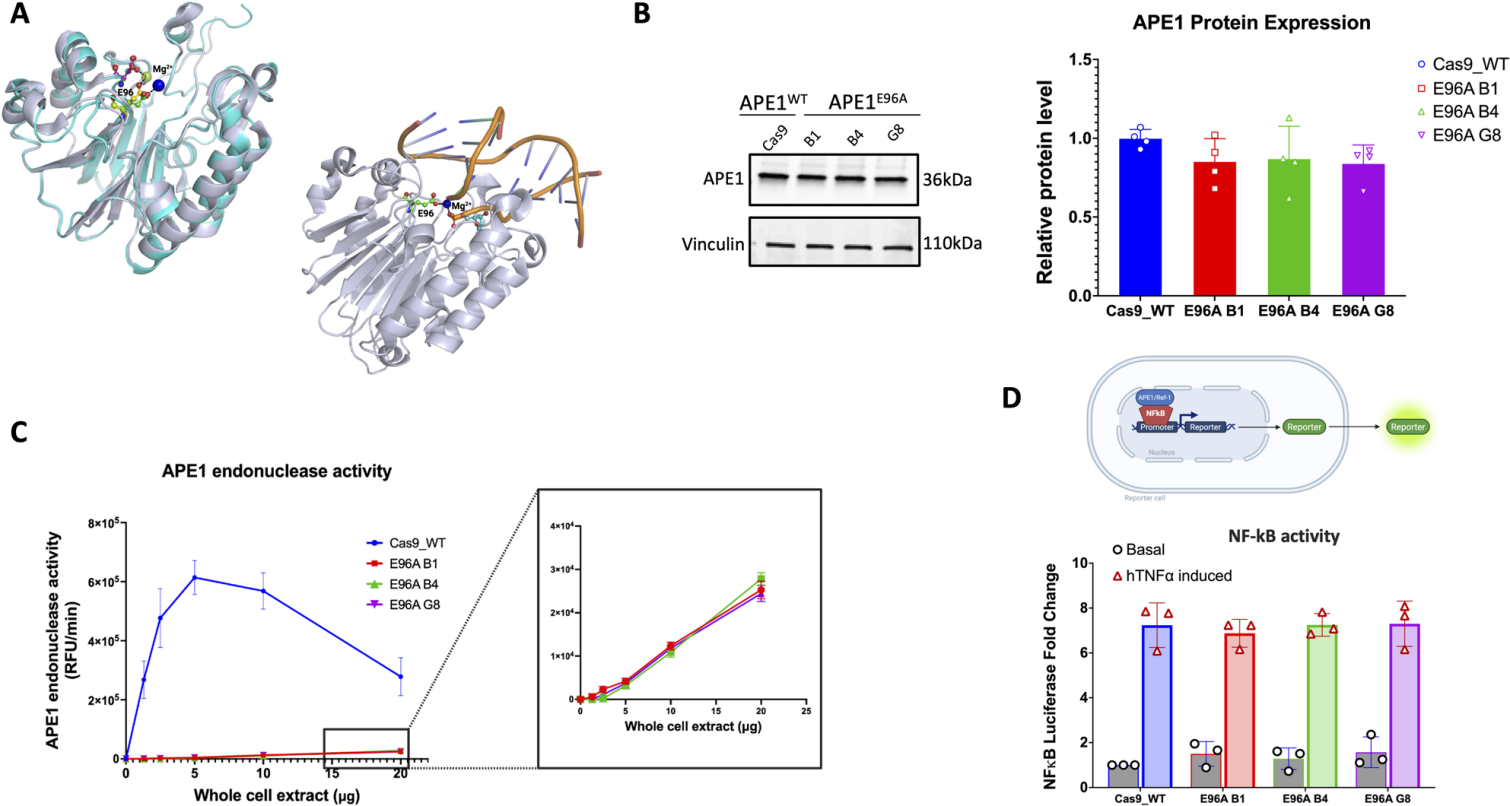
Generation and validation of APE1 endonuclease-deficient PDAC cell models. **A** Glu 96 coordinates Mg^2+^ in structures of APE1 in the presence and absence of bound substrate DNA. In the top-left image, APE1 in the absence of DNA (PDB ID 4QHE) is shown as a cyan cartoon. Mg^2+^ (yellow sphere) is coordinated by both Asp 70 (cyan stick rendering) and Glu 96 (yellow stick rendering). In the presence of DNA, Glu 96 (green stick rendering) coordinates Mg^2+^ (blue sphere) in a slightly altered conformation from that observed in the absence of DNA and positions Mg^2+^ to coordinate the DNA backbone as well (shown in bottom right panel as a light blue cartoon rendering of APE1 and a cartoon rendering of bound DNA (PDB ID 4IEM). **B** Representative Western blot analysis of total APE1 protein levels in Cas9 control and APE1^E96A^ mutant cell lines (B1, B4, G8). Vinculin served as a loading control. A bar graph shows quantification of APE1 abundance (mean ± SEM, *n* = 4; ns, not significant by one-way ANOVA). **C** Real-time fluorescence incision assay comparing endonuclease activity of Cas9 control and APE1^E96A^ mutants. The inset displays a magnified view of the trace activity in E96A clones, which exhibit a ~ 150-fold reduction compared to wild-type. **D** Evaluation of APE1 redox function via NF-κB luciferase reporter assay. Schematic of the reporter system (Top). NF-κB activity in basal and TNFα-stimulated conditions (Bottom). Data represent fold-change over basal (mean ± SEM, *n* = 3). No significant difference was observed between genotypes (*p* > 0.25)

Decades of studies have identified the key residues that facilitate APE1 endonuclease activity [27, 28], including glutamate 96 (E96), which coordinates the Mg^2+^ ion during hydrolytic cleavage [28] (Fig. 1A). E96 enables optimal cleavage but is not absolutely required, making it an attractive target for selectively impairing APE1 endonuclease activity and enabling the study of APE1-directed BER in living PDAC models.

Here, we are the first to report the generation of stable APE1 endonuclease-deficient PDAC cell lines created through a CRISPR/Cas9-mediated homozygous E96A knock-in mutation (APE1^E96A^). This mutation reduces endonuclease activity by approximately 150-fold while maintaining full redox function and normal protein expression levels. These cell lines establish a unique model for studying APE1 endonuclease activity in PDAC cells, including its contributions to genomic maintenance, growth dynamics, and cellular homeostasis. They allow for the direct assessment of PDAC cell reliance on APE1-mediated BER and the evaluation of therapeutic strategies that can target that reliance.

## Methods

### Cell culture and generation of APE1^E96A^ mutant cell lines

Low-passage, patient-derived pancreatic PDAC cells, Pa03C, were cultured in Dulbecco’s Modified Eagle Medium (DMEM; Invitrogen, Carlsbad, CA) supplemented with 10% fetal bovine serum (FBS; GeminiBio, W. Sacramento, CA) at 37 °C in a humidified atmosphere containing 5% CO₂. The Pa03C cell line was originally derived from a liver metastatic site in a male PDAC patient [29]. Cell line authentication was confirmed by short tandem repeat (STR) profiling, and all cultures tested negative for mycoplasma throughout the experiments. Cells were maintained under controlled passage conditions and used within ten passages of thawing.

The APE1^E96A^ mutant cell lines are homozygous knock-in clones containing the APEX1 E96A mutation (Glutamate 96 to Alanine). These mutant cell lines were created in a Pa03C background using CRISPR/Cas9 genome editing by Synthego Corporation (Redwood City, CA, USA). The process utilized ribonucleoprotein complexes (RNPs) consisting of the recombinant Cas9 protein and synthetic, chemically modified single-guide RNAs (sgRNAs). These were electroporated into Pa03C cells along with a single-stranded oligodeoxynucleotide (ssODN) donor template, according to a proprietary Synthego-optimized protocol. Editing efficiency was evaluated 48 h after electroporation. Genomic DNA was extracted from a portion of the cell population, amplified by PCR, and analyzed using Sanger sequencing. The resulting chromatograms were interpreted with the ICE (Inference of CRISPR Edits) analysis tool (Synthego).

For clonal isolation, edited cell pools were seeded at one cell per well using a single-cell printer into 96- or 384-well plates. Wells were imaged every 3 days to verify outgrowth from single-cell origins. Clonal populations were screened and genotyped using PCR followed by Sanger sequencing and ICE analysis. Three independent, homozygous APE1^E96A^ mutant clones were isolated: Pa03C APE1^E96A^ B1 (E96A B1), Pa03C APE1^E96A^ B4 (E96A B4), and Pa03C APE1^E96A^ G8 (E96A G8). A mock control line (Pa03C APE1^WT^, E96A Cas9, or wild-type control) was created by electroporating parental Pa03C cells with the Cas9 protein and donor template in the absence of a sgRNA. Guide RNA and donor sequences are provided in Supplemental Table 1.

### Drug treatment and cytotoxicity

Pa03C cells were seeded at a density of 2,500 cells per well in clear-bottom 96-well plates and allowed to adhere overnight. The next day, cells were treated with methyl methanesulfonate (MMS; Sigma-Aldrich, Cat# 129925), hydrogen peroxide (H₂O₂; Sigma-Aldrich, Cat# H1009), or menadione (Sigma-Aldrich, Cat# M5625). Twenty-four hours after treatment, 10% (v/v) Alamar Blue reagent (Invitrogen, Eugene, OR, USA) was added directly to the culture medium, and the plates were incubated for an additional 4 h at 37 °C. Fluorescence was then measured using a plate reader (excitation: 560 nm, emission: 590 nm). Cell viability was assessed by comparing fluorescence intensity in treated wells to untreated (media-only or vehicle) controls. All conditions were tested in at least triplicate wells, and each experiment was independently repeated at least three times.

### APE1 endonuclease activity assay

A fluorescence-based repair assay was used to quantitatively measure AP site incision. This assay measures APE1-mediated cleavage of a FRET (fluorescence resonance energy transfer) substrate, in which a 5′-fluorescein (6-FAM)-labeled oligonucleotide containing a tetrahydrofuran (THF) abasic site analog is annealed to a complementary strand carrying a 3′-Dabcyl quencher. The sequences were as follows:

$$5^{\prime}-\left(\mathrm{6-FAM}\right)\text{GAA TCC}^{\ast}\text{CCA TAC GTA TTA TAT CCA ATT CC-3}^\prime$$

$$5^{\prime}-\text{GGA ATT GGA TAT AAT ACG TAT GGT GGA TTC}-\left(\mathrm{DABCYL}\right)-3^\prime$$

In the intact substrate, 6-FAM fluorescence is quenched by Dabcyl. Upon incision at the AP site by active APE1, the fluorophore and quencher physically separate, yielding a fluorescence increase directly proportional to APE1 incision activity.

Whole-cell extracts were prepared from Cas9 control cells and three independent E96A mutant clones (B1, B4, and G8). Cells were collected by trypsinization, washed in cold PBS, and lysed by sonication in PBS supplemented with 2 mM DTT. Following centrifugation at 13,000 rpm for 15 min at 4 °C, supernatants were collected, and total protein concentration was determined via Bradford assay. Extracts were aliquoted and stored at -80 °C. To account for differential APE1 expression in the E96A mutant cells, whole cell extract input was normalized to specific APE1 protein levels. The assay was performed in clear-bottom 96-well plates with a total reaction volume of 200 µL per well. Reactions contained 1x assay buffer (50 mM Tris, 1 mM MgCl_2_, 50 mM NaCl, pH 7.5), 50 nM annealed oligonucleotide substrate, and a titrated input of the normalized whole cell extract ranging from 1.25 µg to 20 µg per well. Negative control reactions included a no-extract blank to account for background fluorescence, and reactions supplemented with 50 mM EDTA to inhibit Mg^2+^-dependent APE1 activity. Fluorescence was measured kinetically at 1-minute intervals over 5 min at 37 °C using a BioTek Synergy plate reader (Agilent Technologies) set to 485 nm excitation and 535 nm emission. Endonuclease activity was quantified by calculating the initial reaction rate (expressed as RFU/min) derived from the linear portion of the fluorescence curve. All samples were assayed in technical triplicate across at least three independent experiments. Statistical differences in activity were evaluated using a one-way ANOVA.

### NF-kB luciferase reporter assay

Pa03C APE1^WT^ Cas9 or APE1^E96A^ mutant cells were co-transfected with constructs containing luciferase driven by NF-κB and a Renilla luciferase control, pRL-TK (Promega Corp., Madison, WI), at a 3:1 ratio by using Lipofectamine 2000 (Invitrogen) as previously described [30]. Sixteen hours after transfection, the transfection media was exchanged for regular growth media. For NF-κB-transfected cells, 20 ng/mL hTNFα (R&D Systems, Cat#10291-TA-020) was used for a 6 h induction period, followed by a luciferase activity assay. Firefly and Renilla luciferase activities were assayed by using the Dual Luciferase Reporter Assay System (Promega Corp, Cat# E1910). A lipofectamine-only control was included for each experiment, and values were normalized both to Renilla luciferase to control for cell killing and to the Cas9-uninduced control to calculate fold change in relative luciferase units. All transfection experiments were conducted in triplicate and repeated at least three times in independent experiments.

### Colony formation assays

Pa03C cells were seeded at low density (10,000 cells per 10 cm dish) to ensure that colonies originated from individual cells. After plating, dishes were left at room temperature for approximately 20 min before transferring to the incubator. After cells are allowed to adhere overnight in the incubator, they are treated the following day (450 µM MMS or 15 µM H₂O₂ for 30 min). Doses were chosen to induce measurable DNA damage while maintaining enough cell viability for colony outgrowth. After treatment, cells were washed with PBS and cultured in fresh growth medium for 8–10 days to allow colony development. At the end of the growth period, cells were fixed with methanol and stained with 0.5% methylene blue. Plates were washed three times with deionized H_2_O and then imaged using a ChemiDoc imaging system (Bio-Rad Laboratories, Hercules, California, USA). Clonogenic growth was quantified using ImageJ with the “Colony Area” plugin [31]. The clonogenic capacity of treated cells was normalized to untreated (media-only) controls.

### Quantitative real-time PCR (qRT-PCR)

Total RNA was extracted using the RNeasy Mini Kit (Qiagen, Hilden, Germany) according to the manufacturer’s protocol. RNA concentration and purity were assessed using a NanoDrop spectrophotometer (Thermo Fisher Scientific, MA, USA). This RNA was then used for cDNA synthesis, in which 1 µg of total RNA was reverse-transcribed in a 25 µL reaction volume using the High-Capacity cDNA Reverse Transcription Kit (Applied Biosystems, Warrington, UK).

With the resultant cDNA, quantitative real-time PCR (qRT-PCR) was performed in 96-well plates using SYBR Green PCR Master Mix (Applied Biosystems, Foster City, CA, USA) with a final reaction volume of 20 µL per well. Reactions were run on the CFX96 Real-Time PCR Detection System (Bio-Rad Laboratories, Hercules, California, USA). Gene-specific primers were obtained from OriGene Technologies (Rockville, MD, USA), and primer sequences are listed in Supplemental Table 4. qRT-PCR thermal cycling conditions were as follows: 1 min at 95 °C, followed by 40 cycles of 15 s at 95 °C and 1 min at 60 °C. Relative gene expression was calculated using the 2^−ΔΔCt^ method, with β-actin serving as the internal normalization control.

### Western blot analysis

Cells were lysed in 1% SDS extraction buffer supplemented with protease inhibitors (Santa Cruz Biotechnology, TX, USA). Lysates were sonicated (5 pulses per cycle, 4 cycles), then were heated at 95 °C for 5 min to shear genomic DNA, as previously described. Protein samples were denatured and resolved by SDS-PAGE, then transferred to nitrocellulose or polyvinylidene fluoride (PVDF) membranes via electrophoretic transfer. Membranes were blocked for 1 h at room temperature in 5% (w/v) non-fat dry milk (Bio-Rad Laboratories, Hercules, California, USA) prepared in Tris-buffered saline with 0.05% (v/v) Tween-20 (TBST; Boston BioProducts, MA, USA; Tween-20 from Thermo Fisher Scientific, MA, USA). Membranes were incubated overnight at 4 °C with primary antibodies, followed by a 1-hour incubation with either horseradish peroxidase (HRP)-conjugated secondary antibodies or IR dye secondary antibody. Signal was detected using the ChemiDoc imaging system, and band intensities were quantified using Image Lab (Bio-Rad Laboratories, Hercules, California, USA). All antibodies and catalogue numbers are provided in Supplemental Table 3.

### Bioinformatic analysis and RNA-sequencing

Total RNA was extracted from Pa03C APE1^WT^ Cas9 control and APE1^E96A^ mutant cell lines (B1, B4, G8) using the RNeasy Mini Kit (Qiagen). RNA quantity and purity were assessed by NanoDrop spectrophotometry (Thermo Fisher Scientific, Wilmington, DE, USA), and integrity was verified by the Indiana University School of Medicine Medical Genomics Core. Sequencing libraries were prepared using the Illumina TruSeq Stranded mRNA kit and sequenced on an Illumina NovaSeq 6000 platform (paired-end, ≥ 30 million reads/sample).

RNA-seq data were processed by the Indiana University Collaborative Core for Cancer Bioinformatics (C3B). Reads were aligned to the human genome (hg38) using STAR (v2.7.11b; --outSAMmapqUnique 60) and quantified to GENCODE v47 genes with featureCounts (v2.0.1; -s 2 -p -Q 10 -O). Genes with > 10 reads in ≥ 3 samples were retained, normalized by the TMM method, and analyzed for differential expression using edgeR (v4.6.3; FDR < 0.05). Gene Ontology and KEGG enrichment were assessed with DAVID.

Downstream statistical analyses and visualizations were performed in R (v4.3.1) using ggplot2, pheatmap, EnhancedVolcano, ComplexHeatmap, and clusterProfiler. For heatmap visualizations, color scales were saturated at a log2 fold change of ± 4 to preserve visual contrast across clones with varying degrees of expression magnitude. GSEA was conducted with clusterProfiler using MSigDB Hallmark and KEGG gene sets. To resolve redundancy among overlapping gene sets and facilitate high-level phenotypic comparison, enriched terms were consolidated into ten “Biological Buckets” based on semantic similarity and relevance to PDAC pathophysiology: DNA Repair, Cell Cycle, Immune Response, Cytokine Signaling, Antigen Presentation, ECM/Adhesion, Metabolism, Oxidative Stress, Hypoxia, and Apoptosis. A quantitative Net Enrichment Score was calculated for each bucket as the difference between the -log_10_(FDR) values of the top upregulated and downregulated terms. Scores were capped at ± 10 to mitigate the influence of outlier p-values, providing a bounded composite metric of directional pathway bias.

### Orthotopic mouse model of pancreatic cancer and assessment of metastasis

NSG (NOD.Cg-Prkdc^scid^Il2rg^tm1Wjl^/SzJ) immunodeficient mice were obtained from the Preclinical Modeling and Therapeutics Core of the Indiana University Simon Comprehensive Cancer Center and maintained under pathogen-free conditions. All procedures were conducted in accordance with NIH guidelines and approved by the Institutional Animal Care and Use Committee (IACUC) at Indiana University School of Medicine.

For orthotopic implantation, each mouse received 1.3 × 10⁴ Pa03C pancreatic cancer cells, either the APE1^WT^ Cas9 control or the APE1^E96A^ mutant cell line. Cells were implanted directly into the pancreas under sterile conditions using standard microsurgical techniques. Following implantation, mice were monitored daily and allowed to develop tumors over a five-week period. For the treatment study, after cellular implantation, tumors were allowed to grow for 1 week before treatment. Temozolomide (TMZ) was suspended in a vehicle consisting of 0.5% (w/v) hydroxypropyl methylcellulose and 0.5% (v/v) polysorbate 80 in 100 mM citrate buffer (pH 3.0) and administered via oral gavage at doses of 44 mg/kg or 66 mg/kg [32]. Control animals received an equivalent volume of the vehicle only. Treatment consisted of three 5-day cycles. At the study endpoint, animals were euthanized, and primary tumors, livers, and lungs were collected for further analysis. To ensure a representative sampling of tumor architecture and metastatic burden, each tumor was bisected. One half was flash frozen in liquid nitrogen for molecular analysis, and the other half was fixed in 10% neutral-buffered formalin for histological examination. The left lobe of the liver and both lungs were also formalin fixed. All fixed tissues were processed and stained with hematoxylin and eosin (H&E) for morphological assessment. Quantification of metastatic lesions in the liver and lungs was performed using the HALO™ image analysis platform (Indica Labs), which employs machine learning classifiers trained to identify metastatic foci based on histological features. Digital slide scans were analyzed to calculate the total area of metastatic lesions in each organ, expressed as a percentage of the total tissue area examined [33].

### Mitochondrial DNA (mtDNA) damage calculation

mtDNA damage was quantified in wild-type and E96A Pa03C cell lines using the RayBio^®^ Human Mitochondrial DNA Damage Quantification Kit (Catalog #MTH-DQ), a TaqMan™-based qPCR assay specifically designed to measure lesions in the D-loop region of mtDNA. The assay is based on the principle that DNA lesions impede DNA polymerase progression, resulting in delayed or reduced amplification; thus, greater mtDNA damage correlates with higher quantification cycle (Cq) values [34].

Cells were treated with 50 µM hydrogen peroxide for 1 h to induce oxidative stress. Following treatment, genomic DNA was extracted using the Qiagen DNeasy Blood & Tissue Kit (Qiagen, Hilden, Germany) according to the manufacturer’s protocol. DNA concentration and purity were assessed via fluorometric quantification, and samples were normalized to 5 ng/µL. For each qPCR reaction, 2 µL of DNA (10 ng total) was used. qPCR reactions were assembled using the provided 2X Probe qPCR Master Mix and Primer/Probe Mix, which target both short and long regions of mtDNA. Control reactions containing nuclease-free water were included to validate assay performance. All reactions were performed in technical triplicate and run on a QuantStudio 5 Real-Time PCR System (Applied Biosystems) with the following cycling conditions: 50 °C for 2 min (carryover decontamination), 95 °C for 10 min (initial denaturation), followed by 40 cycles of 95 °C for 10 seconds and 60 °C for 60 seconds.

mtDNA damage was calculated using the comparative ΔΔCq method. The amplification ratio between treated and untreated samples was converted to average lesions per amplicon using the Poisson distribution, with the probability of observing zero lesions given by 𝑒^−𝜆^. Lesions per amplicon were calculated as: λ = ΔΔCq×ln [2]. Values were then normalized to a standard length of 10 kb DNA by multiplying by 10/0.895, where 0.895 kb is the manufacturer-provided amplicon length. Final values were expressed as lesions per 10 kb DNA.

### Annexin V/Propidium Iodide (PI) apoptosis assay

Apoptosis was quantified by Annexin V-FITC/PI dual staining and flow cytometry. Cells were seeded in 6-well plates, treated, and collected as indicated. At the end of treatment, both adherent and floating cells were collected, washed once with cold phosphate-buffered saline (PBS) and once with cold 1X binding buffer, and resuspended in 100 µL of 1X binding buffer. For each condition, 1 × 10⁶ cells were incubated with 10 µL of Annexin V-FITC (BioLegend, San Diego, CA, USA) at room temperature in the dark for 15 min. Cells were then washed with 1 mL of 1X binding buffer and resuspended in 200 µL of 1X binding buffer containing 5 µL of propidium iodide (Thermo Fisher Scientific, Waltham, MA, USA), following the manufacturer’s protocol.

Samples were analyzed within 1 h using an Attune NxT flow cytometer at the IUSCCC Flow Cytometry Core. Data were acquired with Attune NxT software and analyzed using FlowJo v10 (BD Biosciences). Apoptotic populations were gated as follows: Annexin V⁻/PI⁻ (viable), Annexin V⁺/PI⁻ (early apoptosis), Annexin V⁺/PI⁺ (late apoptosis/secondary necrosis), and Annexin V⁻/PI⁺ (primary necrosis). At least 10,000 singlet events were collected per sample. Results are expressed as percentages of early, late, and total apoptotic cells, with experiments performed in triplicate. For quantification of total cell death, compromised cells were calculated by combining AnnexinV-positive/PI-negative (early apoptotic), AnnexinV-positive/PI-positive (late apoptotic), and AnnexinV-negative/PI-positive (membrane-compromised) populations. Statistical comparisons of total cell death were performed on this combined metric to capture overall cell death and membrane compromise across all measured modalities.

### MicroRNA calculation

#### Sample collection and preparation

Blood samples were collected from mice at multiple time points (weeks 1, 3, and 5 post-implantation). Plasma was isolated by centrifugation and stored at -80 °C in RNase-free tubes.

##### Fabrication of the nanoplasmonic biosensing platform

The nanoplasmonic biosensing platform was fabricated for the detection of microRNAs in mouse plasma using a previously reported procedure with minor modifications [35]. In the fabrication strategy, gold triangular nanoprisms (Au TNPs) were functionalized with spiropyran (SP)-derived self-assembled monolayers (SAMs).

##### Synthesis and immobilization of Au TNPs

Au TNPs were synthesized following established protocol [36]. Next, glass coverslips were silanized according to a previously published method [37]. Silanized glass coverslips were incubated in the freshly synthesized Au TNP solution for 1 h, then rinsed thoroughly with acetonitrile and dried under a gentle stream of nitrogen. Glass substrates bearing attached Au TNPs were stored in a glass container under nitrogen at 4 °C until further use. The successful immobilization of AuTNPs onto glass surfaces was verified by monitoring the localized surface plasmon resonance (LSPR) peak (λ_LSPR_) using UV-visible spectroscopy. The Au TNP-attached coverslips were then attached to the bottom of a 96-well bottomless microplate using a minimal amount of cyanoacrylate adhesive and allowed to cure for 2 h at room temperature [38]. Attachment was confirmed by measuring the λ_LSPR_ of Au TNPs using a microplate reader in the absorbance mode.

##### Au TNP surface functionalization with spiropyran-hexanethiol: hexanethiol (SP-HT: HT)

The fabrication of a nanoplasmonic biosensing platform is a two-step process. First, silanized glass coverslip-bound Au TNPs in a 96-well plate format were functionalized by incubating in a mixture of 0.025 M SP-HT and 0.025 M HT of a 75:25% mole ratio for overnight to form a mixed SAM. Next, the well plates were rinsed extensively with acetonitrile to remove any excess and loosely bound thiols, and then dried under nitrogen. The mixed SP-HT: HT SAM formation was verified by monitoring the λ_LSPR_ of Au TNPs. Second, mixed SAM modified Au TNPs in a 96-well plate were irradiated with 370 nm UV light for 5 min 30s using Kessil photoreaction lamps (model KSPR160L-370, operated at 50% power) [39]. The successful conversion of SP to the zwitterionic MC state was confirmed by a characteristic λ_LSPR_ red shift of Au TNPs.

##### Capture probe immobilization and microRNA detection

96-well plate containing MC-HT -functionalized Au TNPs was incubated in 0.3 mL of 10.0 µM single-stranded DNA (-ssDNA-X, X = 10b, 155) probes solution (7.2 pH PBS buffer) specific to either microRNA-10b (ssDNA-10b) or microRNA-155 (ssDNA-155) overnight. The next day, each well was thoroughly rinsed with PBS, and successful probe immobilization was confirmed by monitoring the λ_LSPR_ using a plate reader. The immobilization of -ssDNA-X probes onto MC-HT: HT SAMs produced a nanoplasmonic biosensing platform for microRNA detection. Here, each well is programmed to detect either microRNA-10b or -155, depending on the -ssDNA-X probe used during fabrication. To detect microRNA-10b, 7.5 µL of mouse plasma was diluted with 220 µL of PBS buffer, vortexed, and then transferred to a single well containing -ssDNA-10b capture probes. An identical process was followed for the detection of microRNA-155. Wells containing diluted mouse plasma were incubated overnight to capture as many microRNAs as possible. After incubation, the wells were extensively rinsed with PBS to remove any non-specifically bound biomolecules. The optical shift (Δλ_LSPR_) was determined as the difference between the λ_LSPR_ value obtained due to the hybridization of target miRNAs with -ssDNA-X probes.

## Results

### E96A mutation markedly diminishes APE1 endonuclease activity without affecting redox signaling

To validate the APE1^E96A^ mutant cell lines, we first examined APE1 protein expression. Because genetic alteration of APE1 can lead to compensatory changes in its expression [25], we performed Western blot analysis. We observed comparable APE1 protein levels between Cas9 control and E96A mutant cell lines (Fig. 1B).

We then assessed APE1 endonuclease activity directly using a quantitative fluorescence-based repair assay. We observed a very dramatic and significant reduction in enzymatic activity in all three APE1^E96A^ mutant cell lines which retained only 0.6–0.8% of incision activity (Fig. 1C). Individually, this corresponds to activity reductions of 133-fold (E96A B1), 170-fold (E96A B4), and 145-fold (E96A G8) relative to the Cas9 control, with an average 150-fold decrease in endonuclease function.

APE1 endonuclease activity operates independently of its redox signaling [25], making the E96A mutation unlikely to influence APE1’s activation of target transcription factors. To confirm this, we tested APE1 redox signaling with a luciferase-based reporter assay. We found that the E96A mutant cell lines fully activated NF-kB, a transcription factor under APE1 redox regulation, following TNFα induction, with no differences relative to the Cas9 control (Fig. 1D).

These results demonstrate that the APE1^E96A^ mutant cell lines exhibit significantly reduced endonuclease activity, with no compensatory changes in APE1 protein levels or alterations in redox signaling activity. 

### E96A mutants exhibit long-term, not acute, sensitivity to genotoxic stress

**Fig. 2:**
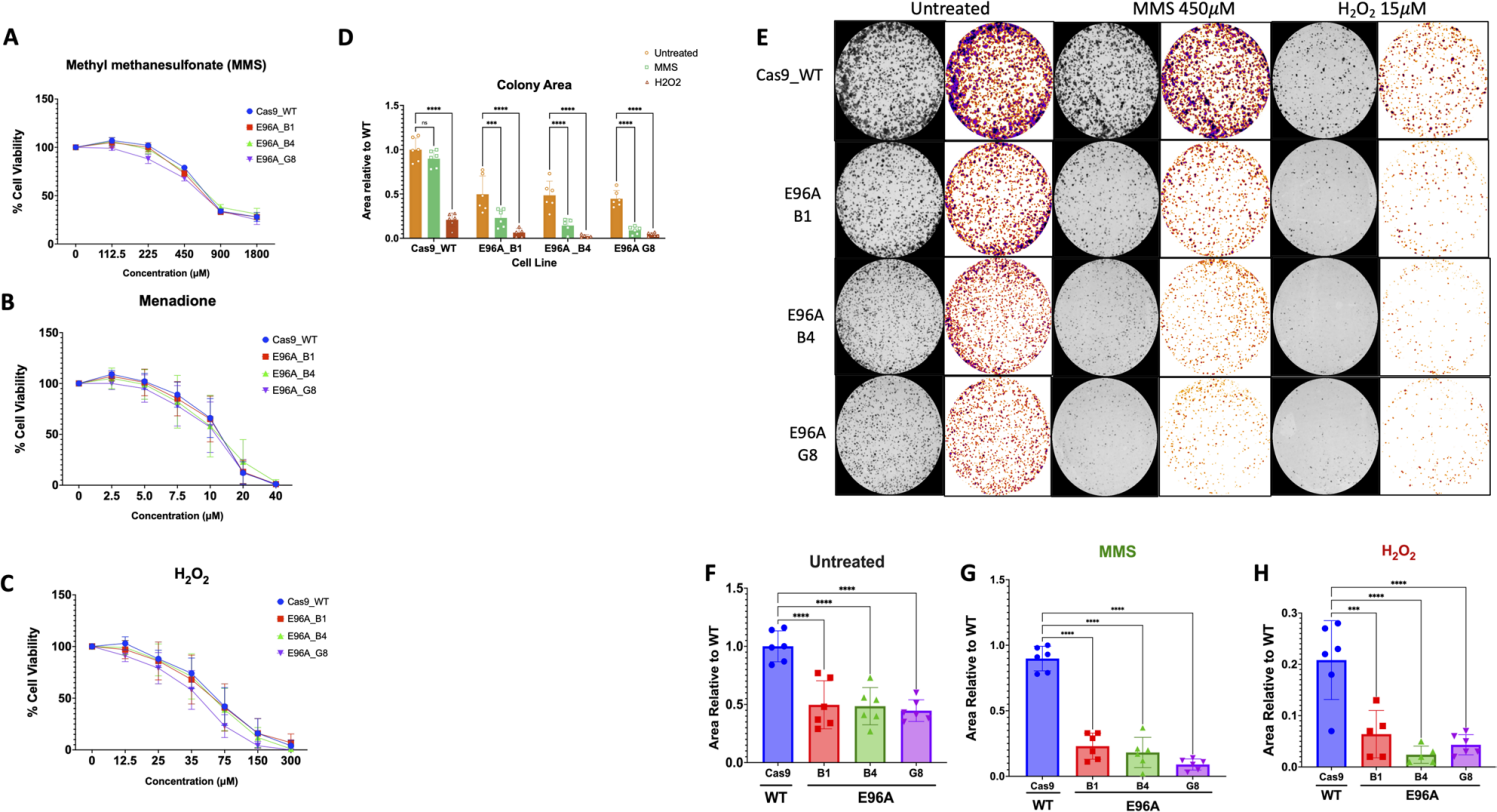
Short-term cytotoxicity assays mask genotoxic sensitivity in E96A mutant cell lines, which is uncovered by long-term colony-forming assays. **A**-**C** Short-term viability dose-response curves. Cells were treated with increasing concentrations of (A) Methyl methanesulfonate (MMS), **B** Menadione, or (**C**) H_2_O_2_ for 24 h. Viability was assessed relative to vehicle control. Note the overlapping cytotoxicity profiles between Cas9 and E96A lines. **D** Aggregated quantification of colony area across all treatment conditions. **E** Representative binary masks derived from methylene blue-stained colonies following mock treatment (Untreated) or a 30-min pulse exposure to MMS (450 µM) or H_2_O_2_ (15 µM) followed by recovery. **F**-**H** Quantification of colony area normalized to WT (Cas9) controls for (**F**) untreated cells, (**G**) MMS-treated cells, and (**H**) H_2_O_2_-treated cells. Comparison of short-term continuous exposure (**A**-**C**) versus acute recovery (**E**-**H**) reveals a defect specific to clonogenic potential. Data represent mean ± SEM (*n* = 3); ***p < 0.001, ****p < 0.0001 by one-way ANOVA with Tukey’s post-hoc test

To investigate the cellular effects of decreased APE1 endonuclease activity, we assessed the acute sensitivity of E96A mutant cell lines to genotoxic stress using the alkylating agent MMS and the oxidizing agents H₂O₂ and menadione (a redox-cycling quinone) in short-term cytotoxicity assays. Across all agents, the E96A mutant cell lines showed dose-response profiles similar to those of the Cas9 control (Fig. 2A-C).

To assess the long-term proliferative capacity of the cell lines, we performed colony-forming assays (Fig. 2D-H). Even without drug treatment, the E96A mutation reduced colony-forming efficiency by 50% compared to the Cas9 control (Fig. 2F). Following 30 min of MMS exposure at 450 µM, Cas9 control cells retained 90% of their colony-forming ability over the study period, whereas the E96A mutant cell lines averaged only about 15–25%, a 70–85% relative reduction compared to the control (Fig. 2G). H₂O₂ (15 µM) showed a similar response; Cas9 cells formed 21% of their original colonies following treatment, while E96A clones formed only 2–6%, a 70–90% relative reduction (Fig. 2H). These results suggest that although E96A-expressing cells initially cope with baseline DNA damage, they are unable to maintain genomic integrity under genotoxic stress, leading to diminished long-term proliferative capacity.

### E96A cell lines register elevated mitochondrial and nuclear DNA damage following oxidative stress

To determine whether APE1 repair activity is required for nuclear genome integrity, we first assessed phosphorylated histone H2AX (γH2AX), a well-established marker of DNA damage and double-strand breaks. For these experiments, we utilized APE1^E96A^ G8 as the representative mutant cell line, as it expressed APE1 levels equivalent to the other APE1^E96A^ mutants and exhibited a stable and reproducible phenotype.

**Fig. 3:**
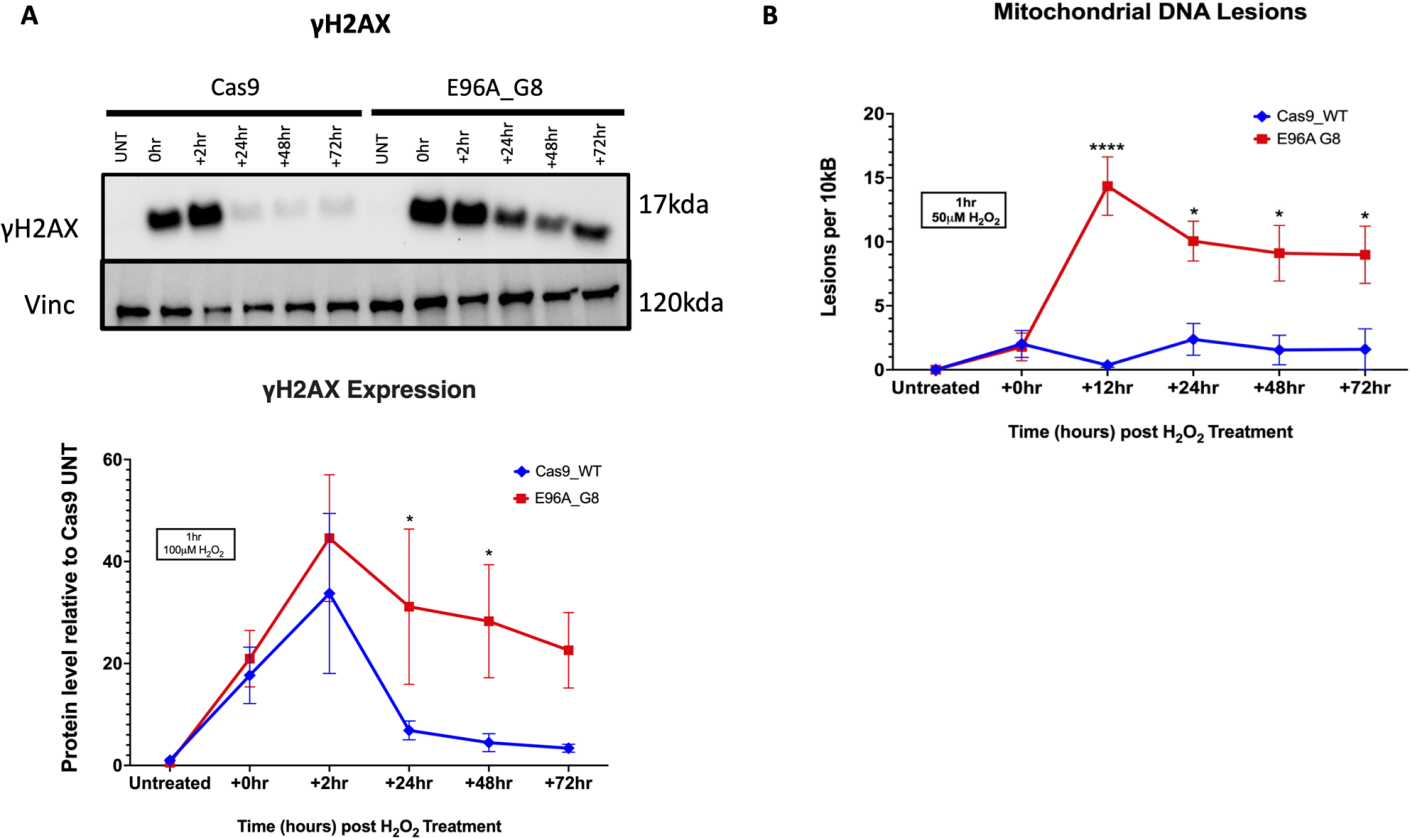
E96A cell lines exhibit increased mitochondrial and nuclear DNA damage following oxidative stress. **A** Sustained nuclear DNA damage response. Top: Representative Western blot of γH2AX (17 kDa) and Vinculin (120 kDa; loading control) after treatment with 100 µM H₂O₂ for 1 h. The E96A G8 mutant shows a prolonged γH2AX signal that persists through 72 h post-treatment. Bottom: Densitometric quantification of γH2AX protein levels normalized to the untreated (UNT) Cas9 control. Data represent mean ± SEM (*n* = 3). Significance determined by ANOVA; *P < 0.1. **B** Mitochondrial DNA (mtDNA) lesion frequency. Cells were treated with 50 µM H₂O₂ for 1 h, followed by recovery for the indicated times. Lesion frequency (per 10kb) was quantified via long-amplicon qPCR using the Poisson distribution. E96A G8 cells show increased lesion accumulation peaking at 12 hours compared to Cas9 control. Data represent mean ± SEM (n=3). Significance determined by ANOVA; *P < 0.05, ****P < 0.0001.

Under untreated conditions, both Cas9 control and E96A G8 cells expressed minimal γH2AX levels (Fig. 3A). Following exposure to 100 µM H₂O₂, γH2AX expression increased sharply in both cell lines. However, by 24 h, a significant difference emerged: γH2AX expression levels in Cas9 control cells returned towards baseline, whereas E96A G8 cells retained elevated levels that persisted through 72 hours.

APE1 knockdown has previously been shown to compromise mitochondrial homeostasis, with prior analysis identifying mitochondrial pathways among those disrupted by APE1 suppression [40]. While APE1’s redox signaling function is known to contribute significantly to mitochondrial stability [22, 23], the specific extent to which the loss of its DNA repair capacity drives this disruption remains to be fully elucidated. Because mtDNA lacks protective histones and resides in an oxidative environment, it is uniquely susceptible to unrepaired damage. To determine the role of APE1 repair activity in the repair of mitochondrial DNA, we quantified mtDNA lesion accumulation over time (Fig. 3B). At baseline, mtDNA lesion burden was similar between the E96A and Cas9 control cells, and this persisted after an hour of exposure to 50 µM H₂O₂, with both cell lines exhibiting about 2 lesions per 10 kb on average. By 12 h post-treatment, the lesion load in the E96A cells had risen to 15 lesions per 10 kb, whereas Cas9 cells had repaired almost all damage, with a residual 0.3–0.4 lesions per 10 kb. This 40-fold higher mtDNA damage in the E96A cells persisted over time, and even 72 h after H_2_O_2_ treatment, the repair-deficient cells retained 5-6-fold more mtDNA lesions compared to Cas9 control (Fig. 3B).

These results demonstrate that APE1 endonuclease activity is essential for maintaining genome integrity in both nuclear and mitochondrial compartments, and that its loss leads to accumulation of DNA damage that likely underlies the enhanced sensitivity of these cells to genotoxic agents.

### E96A cell lines exhibit elevated overall cell death following oxidative stress

Given the decreased colony-forming units and the persistent mitochondrial and nuclear DNA damage in E96A cells following genotoxic exposure, we next performed Annexin V/PI flow cytometry to evaluate whether this damage culminates in a specific mechanism of cell death.

**Fig. 4:**
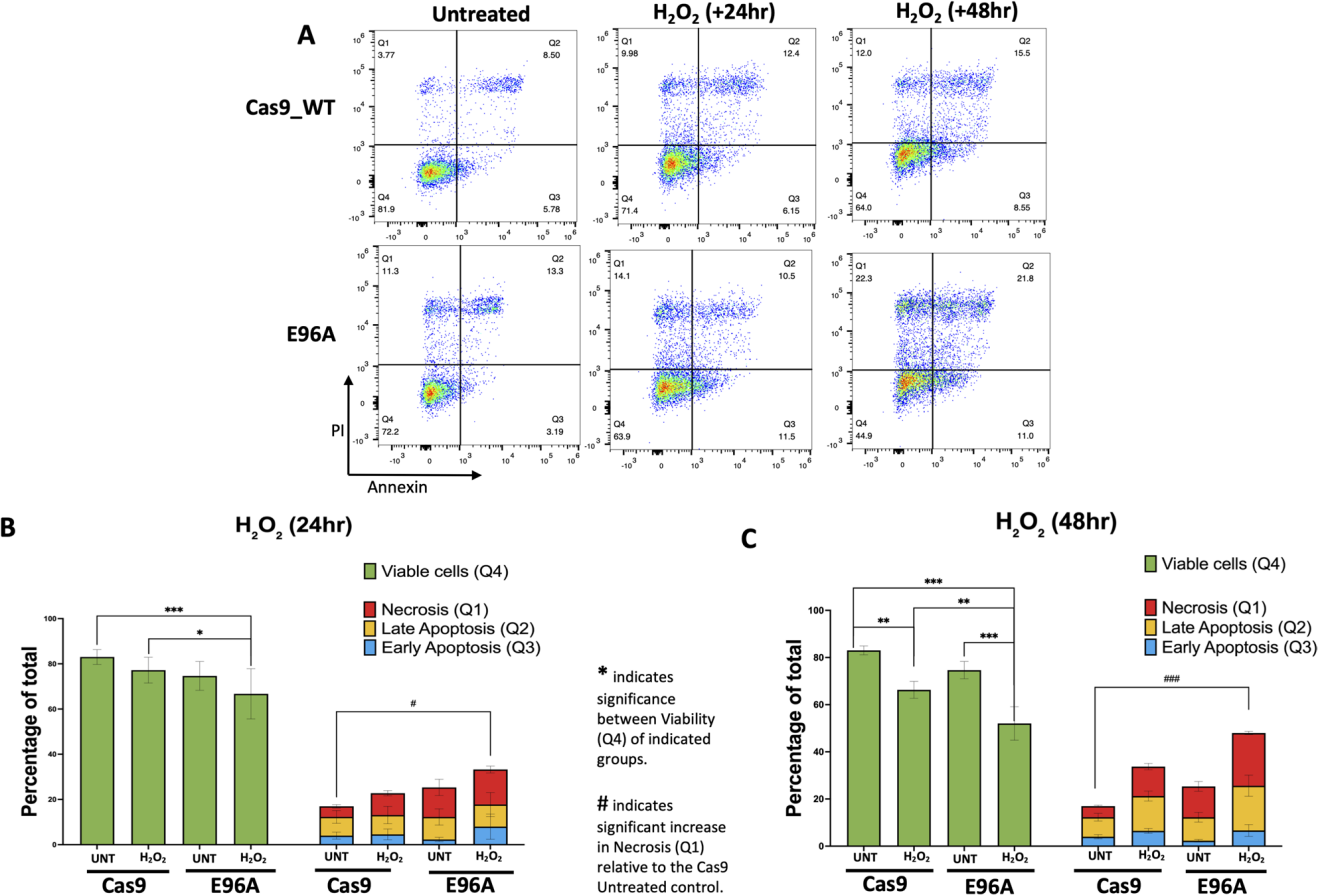
E96A cell lines show increased overall cell death after oxidative stress. **A** Representative flow cytometry plots of Annexin V/Propidium Iodide (PI) staining at 24 and 48 h post-treatment. **B**, **C** Quantification of cell fate distributions at (B) 24 h and (C) 48 h. Stacked bars display the proportion of viable (Q4), early apoptotic (Q3), late apoptotic (Q2), and necrotic (Q1) cells. Statistical significance was determined by two-way ANOVA with Tukey’s multiple comparisons. Asterisks (*) indicate significant differences in cell viability (Q4) between genotypes (*p < 0.05, **p < 0.01, ***p < 0.001). Hashes (#) indicate a significant increase in necrosis (Q1) relative to the untreated Cas9 control (#*p* < 0.05, ###*p* < 0.001)

Oxidative stress (H_2_O_2_) induced a significant increase in total cell death (Q1-Q3) in both genotypes between 24-and 48-hour timepoints (Cas9: 16.9% to 33.7%, *p* = 0.014; E96A: 25.3% to 48.0%, *p* = 0.046). However, the distribution of cell death states differed between genotypes. While apoptotic fractions (Annexin+) showed no significant variance between Cas9 and E96A cells (*p* = 0.56), E96A cells exhibited a pronounced enrichment in the PI+/Annexin- (Q1) population (Fig. 4A-C). 48 h after H_2_O_2_ treatment, the PI+/Annexin- fraction in E96A cells rose to 22.4%, compared to 12.5% in Cas9 controls (*p* = 0.0028). This increase in membrane-compromised cells was the most statistically robust difference observed between genotypes and coincided with a significant reduction in overall viability (*p* = 0.006).

To determine whether this vulnerability extended to alkylating agents, we repeated the assay with MMS (Supplementary Fig. S1). This confirmed the necrotic phenotype with even greater statistical clarity. While Cas9 cells restricted PI+/Annexin- to 8.3% after 48 h of treatment, E96A cells exhibited a near-tripling of the necrotic fraction to 22.8% (*p* = 0.005).

These data indicate that loss of APE1 endonuclease activity is associated with an increased propensity toward membrane integrity failure under oxidative stress. In the context of persistent mitochondrial and nuclear DNA damage, this shift likely contributes to the reduced survival capacity of E96A cells.

### E96A baseline transcriptome shows downregulation of ECM and cell adhesion without activation of compensatory repair mechanisms

**Fig. 5:**
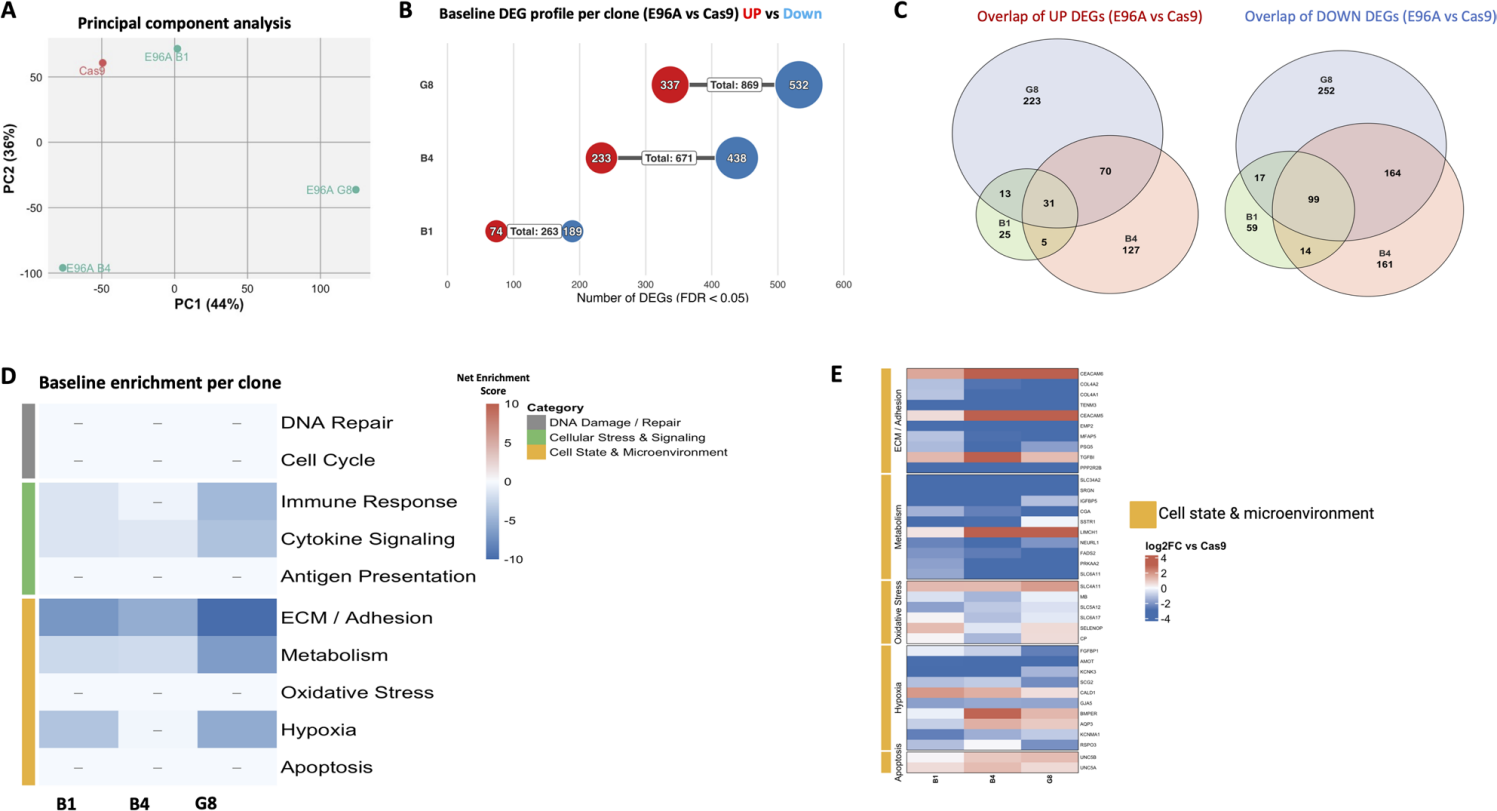
E96A baseline transcriptome shows selective suppression of ECM and adhesion pathways. **A** Principal Component Analysis (PCA) of global gene expression profiles from Cas9 control and E96A mutant clones (*n* = 3). **B** Quantification of differentially expressed genes (DEGs) for each clone relative to Cas9 control (FDR < 0.05, |log2FC| > 1). Red indicates upregulation; blue indicates downregulation. **C** Venn diagrams displaying the intersection of unique and shared DEGs across the three mutant clones for upregulated (left) and downregulated (right) gene sets. **D** Pathway enrichment analysis aggregated into ten functional biological buckets. Heatmap intensity represents the Net Enrichment Score, calculated as the difference between the -log_10_(FDR) of the top upregulated and downregulated terms (capped at ± 10). Red indicates net pathway activation; blue indicates net suppression. Note the absence of significant enrichment in DNA Repair or Cell Cycle buckets (indicated by dashes). **E** Heatmap of log2 fold-change values for representative genes within the Cell State & Microenvironment modules. Blue indicates downregulation relative to Cas9

To examine the baseline transcriptional consequences of deficient APE1 endonuclease activity, we performed RNA sequencing on the E96A mutant cell lines in comparison with the Cas9 control. Principal component analysis (PCA) showed a clear separation of the Cas9 control from the E96A mutants, indicating a distinct genotype-driven transcriptional program associated with loss of APE1 endonuclease activity, though this was accompanied by noticeable inter-clonal heterogeneity (Fig. 5A). This variation was reflected in the burden of differentially expressed genes (DEGs); each mutant clone exhibited a distinct profile of DEGs (FDR < 0.05), with B1 showing 263 DEGs (74 up, 189 down), B4 showing 671 DEGs (233 up, 438 down), and G8 showing 869 DEGs (337 up, 532 down) (Fig. 5B). DEG overlap across clones was limited, with only 31 commonly upregulated and 99 commonly downregulated transcripts (Fig. 5C), indicating clone-specific transcriptomic divergence. 

Despite this quantitative variance, the qualitative functional signature was remarkably conserved. We consolidated enriched terms into broad “biological buckets” to assess the net directional bias of key pathways. Analysis revealed consistent downregulation of extracellular matrix (ECM) and cell adhesion pathways, as well as selected cell-cycle pathways, across all three E96A clones, while upregulated signatures were modest and largely heterogeneous (Fig. 5D).

To visualize the specific drivers of these pathway shifts, we examined the magnitude of expression changes for representative genes within the significantly altered categories (Fig. 5E). The suppression of the ECM program was deep and broad, characterized by the downregulation of structural collagens (COL4A1, COL4A2) with log2-fold changes frequently exceeding − 2.

No significant enrichment of DNA repair or BER-associated pathways was observed among upregulated genes, suggesting an absence of transcriptional compensation for impaired APE1 endonuclease activity.

Collectively, the baseline transcriptome of APE1^E96A^ cells is defined by a consistent suppression of pathways governing cell adhesion and proliferation, without evidence of a compensatory transcriptional response to the DNA repair defect.

### E96A cell lines show decreased tumor growth, metastasis potential, and circulating oncogenic microRNA in vivo

**Fig. 6:**
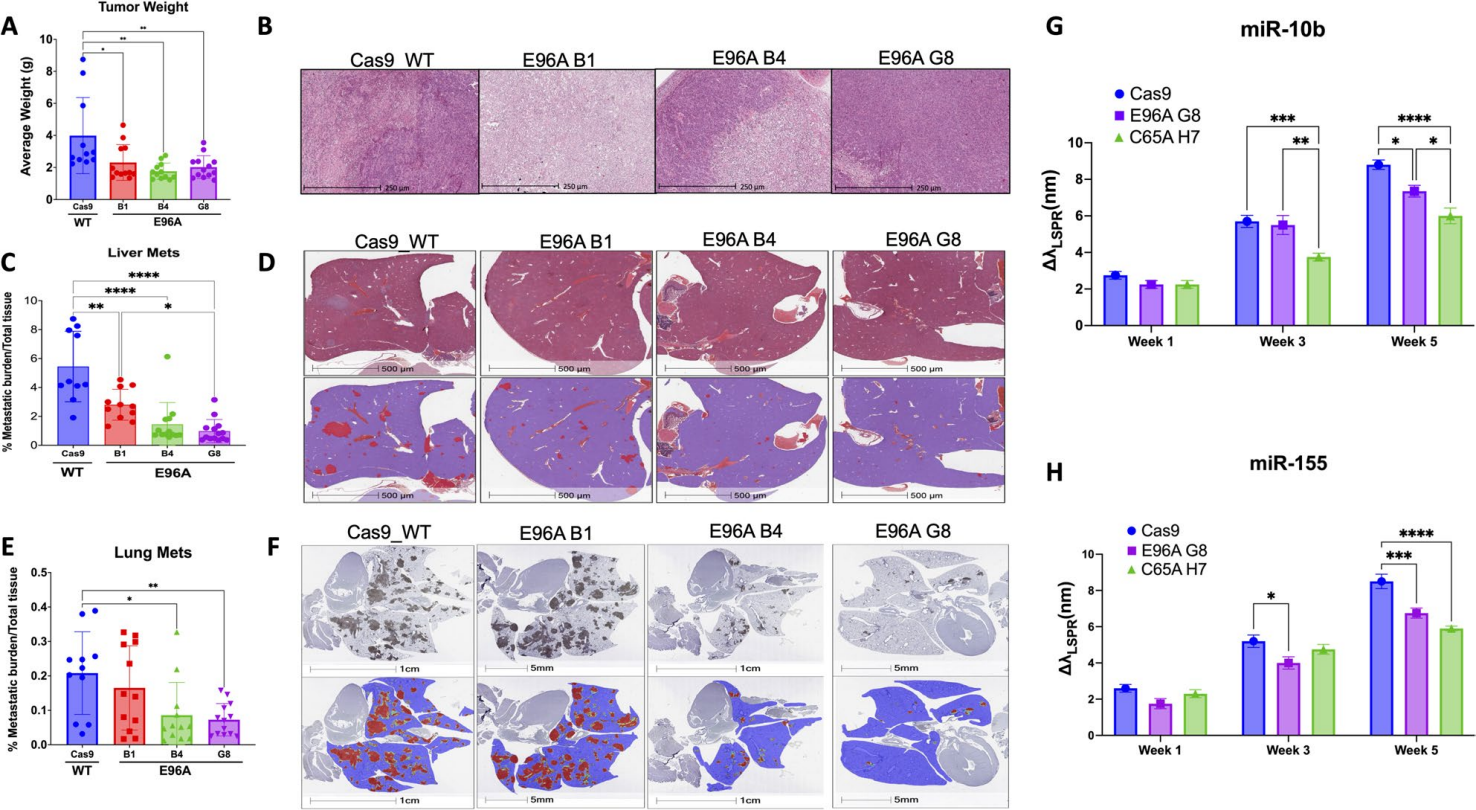
E96A cell lines show decreased tumor growth, metastatic ability, and circulating biomarkers in vivo without treatment. **A** Final weights of primary pancreatic tumors excised 5 weeks post-orthotopic implantation of Cas9 control and E96A mutant lines (*n* = 11–13 mice per group). **B** Representative Hematoxylin and Eosin (H&E) staining of primary tumor tissue. **C** Quantification of hepatic metastatic burden, calculated as the percentage of metastatic tissue area relative to total tissue area. **D** Representative liver histology. (Top) Raw H&E-stained sections. (Bottom) Digital segmentation masks used for burden quantification, where red indicates metastatic foci and blue indicates normal hepatic tissue. **E** Quantification of pulmonary metastatic burden. **F** Representative lung histology (top) and corresponding segmentation masks (bottom). **G**, **H** Longitudinal analysis of circulating miR-10b (**G**) and miR-155 (**H**) levels in plasma at weeks 1, 3, and 5 post-implantation. Data represent the shift in localized surface plasmon resonance peak wavelength (Δλ_LSPR_) relative to baseline. The redox-deficient APE1 C65A mutant (clone H7) is included as a reference for non-endonuclease-mediated suppression. Data represent mean ± SEM (*n* = 6). Statistical significance was determined by one-way ANOVA (A, C, E) or two-way ANOVA (G, H) with Tukey’s multiple comparisons test (*p < 0.05, **p < 0.01, ***p < 0.001, ****p < 0.0001)

Having previously established that APE1 redox function drives PDAC progression using C65A redox-deficient mutants [25], we investigated whether selective impairment of APE1 endonuclease activity similarly affects tumor growth and metastatic potential in vivo. We implanted APE1^WT^ Cas9 control and APE1^E96A^ mutant cells orthotopically into the pancreas of immunocompromised mice. After five weeks of growth without treatment, mice implanted with the three E96A mutant cell lines had significantly lower tumor burden than those implanted with Cas9 control, with an average 50% reduction in primary tumor mass (Fig. 6A-B).

The APE1^E96A^ mutation also suppressed metastasis, although this effect varied by clone. While all three APE1^E96A^ mutants showed significantly reduced liver metastasis, mice implanted with the APE1^E96A^ B1 cell line had a higher metastatic burden than those implanted with APE1^E96A^ B4 or APE1^E96A^ G8 (Fig. 6C-D). Similarly, in lung metastasis, mice implanted with APE1^E96A^ B4 or APE1^E96A^ G8 showed significantly reduced metastasis compared to the APE1^WT^ Cas9 control, whereas mice with APE1^E96A^ B1 did not differ significantly from the control group (Fig. 6E-F). As primary tumor sizes were comparable across all mutant lines, these differences in metastatic burden are independent of primary tumor growth.

To evaluate whether the loss of APE1’s repair function mimics the systemic effects of its redox deficiency, we longitudinally profiled PDAC-specific circulating microRNAs from plasma, focusing on miR-10b and miR-155, two oncogenic microRNAs involved in PDAC invasion and metastasis [41]. This approach allowed for a direct comparison between the endonuclease-deficient mutant (E96A G8) and the redox-deficient mutant (C65A H7). APE1^C65A^ H7 was previously characterized as having lower metastatic potential.

Levels of the biomarkers were measured in plasma from mice bearing Cas9 control, APE1^E96A^ G8, or APE1^C65A^ H7 tumors. At week 3, while miR-155 levels were already significantly reduced in the E96A cohort compared to the Cas9 control (*p* < 0.05), miR-10b levels remained comparable between Cas9 control and E96A tumors but were lower in the C65A tumors, indicating differential temporal regulation. However, by week 5, the loss of endonuclease and redox activity resulted in a distinct suppression of both markers. APE1^E96A^ mice exhibited a 21% reduction in miR-155 (*p* < 0.001) and a 16.5% reduction in miR-10b (*p* < 0.05) compared to controls (Fig. 6G-H). Both APE1 repair deficient and redox deficient tumors impact these circulating biomarkers which correlated well with a decrease in primary tumor size and metastatic burden.

Together, these results demonstrate that APE1 endonuclease deficiency reduces both local tumor fitness and systemic metastatic potential, with a corresponding suppression of circulating oncogenic miRNA biomarkers.

### E96A mutation sensitizes cells to platinum and alkylating chemotherapies

**Fig. 7:**
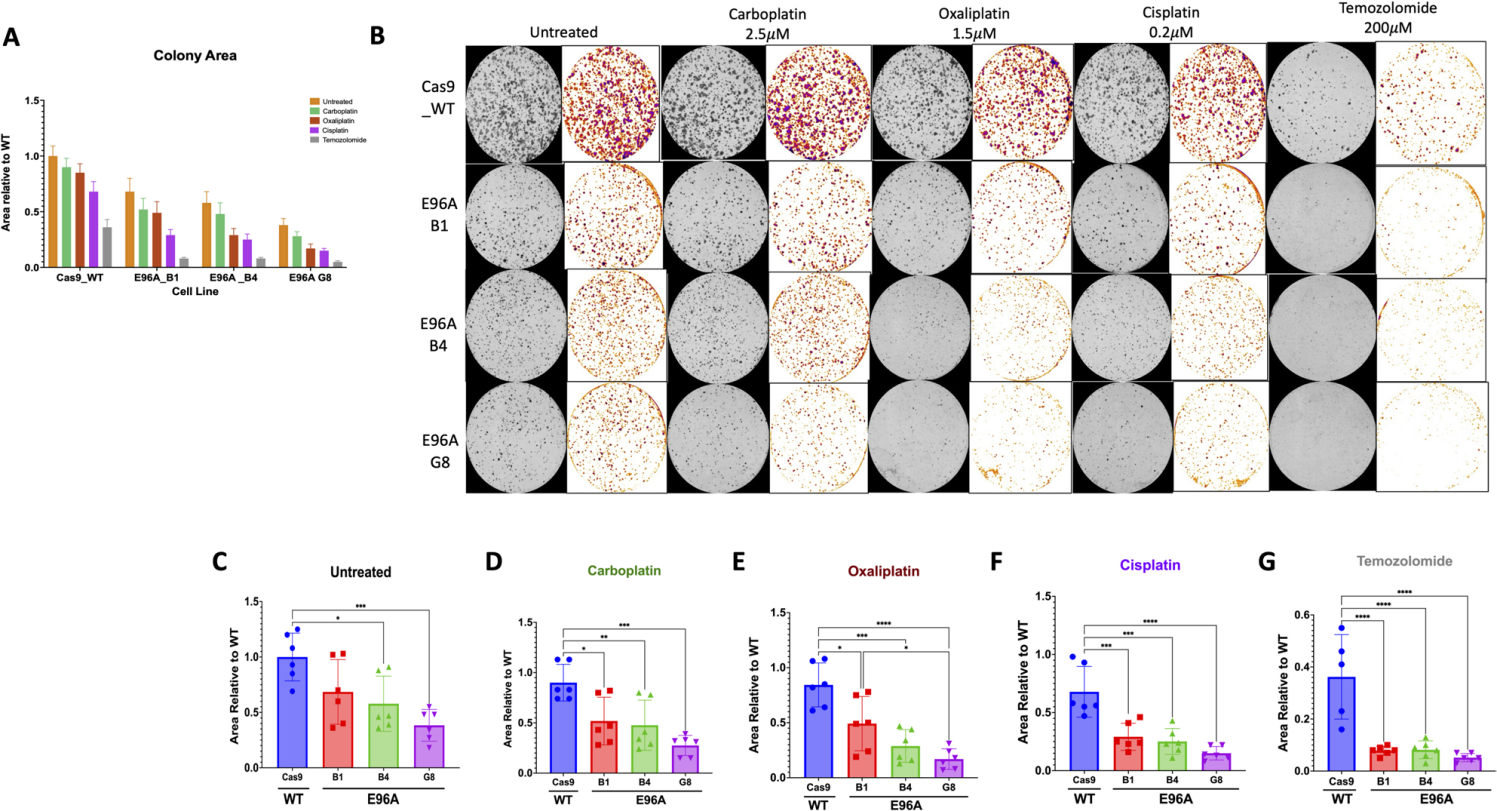
E96A cell lines show decreased growth and proliferation after treatment with platinum and alkylating chemotherapy agents. **A** Aggregated quantification of colony area across all chemotherapeutic treatment conditions. **B** Representative images of methylene blue-stained colonies (grayscale columns) and corresponding binary segmentation masks (colored columns) following long-term recovery from treatment with carboplatin (2.5 µM), oxaliplatin (1.5 µM), cisplatin (0.2 µM), or temozolomide (200 µM). **C**-**G** Quantification of colony area normalized to untreated WT (Cas9) controls for (**C**) Untreated, (**D**) Carboplatin, (**E**) Oxaliplatin, (**F**) Cisplatin, and (**G**) Temozolomide treated cells. Data represent mean ± SEM (*n* = 3). Statistical significance was determined by one-way ANOVA with Tukey’s post-hoc test (*p < 0.05, **p < 0.01, ***p < 0.001, ****p < 0.0001)

Given the observed susceptibility to oxidative and alkylating stress, we hypothesized that APE1^E96A^ mutant cell lines would exhibit similar hypersensitivity to clinically used DNA-damaging agents that generate these kinds of genotoxic stress. To test this, we performed clonogenic formation assays against a panel of chemotherapeutics, including platinum-based drugs (cisplatin, carboplatin, oxaliplatin) and the alkylator temozolomide (TMZ) (Fig. 7A-B).

For the platinum compounds, doses were chosen to cause minimal effects on the Cas9 control cells. Treatment with carboplatin (2.5 µM), oxaliplatin (1.5 µM), or cisplatin (0.2 µM) showed drug sensitivity proportional to each compound’s ability to generate ROS, with the greatest loss of viability seen with cisplatin and TMZ (Fig. 7B-G). The absolute differences between Cas9 (ΔCas9-E96A G8) after treatment were 0.625 for carboplatin (*p* < 0.001), 0.528 for cisplatin (*p* < 0.0005), 0.673 for oxaliplatin (*p* < 0.0001), and 0.310 for TMZ (*p* < 0.0001). When normalized to the Cas9 baseline to show proportional effects, these corresponded to decreases of 69.4%, 77.9%, 79.8%, and 85.7% in colony formation, respectively. The pattern showed a clear rank order of temozolomide > oxaliplatin ≈ cisplatin > carboplatin (Fig. 7C-G). These results demonstrate that APE1 endonuclease deficiency sensitizes PDAC cells to clinically relevant drugs, particularly those that generate lesions requiring BER for their repair.

### Temozolomide treatment enhances the anti-tumor effect of the E96A mutation in vivo

To determine whether the alkylating sensitivity observed in vitro translates into therapeutic efficacy in vivo, we again used the orthotopic mouse model. Mice were implanted with either Cas9 control or APE1^E96A^ G8 cells and treated with vehicle or TMZ. We selected doses of 44 mg/kg and 66 mg/kg to approximate clinically relevant human doses [42–44].

**Fig. 8:**
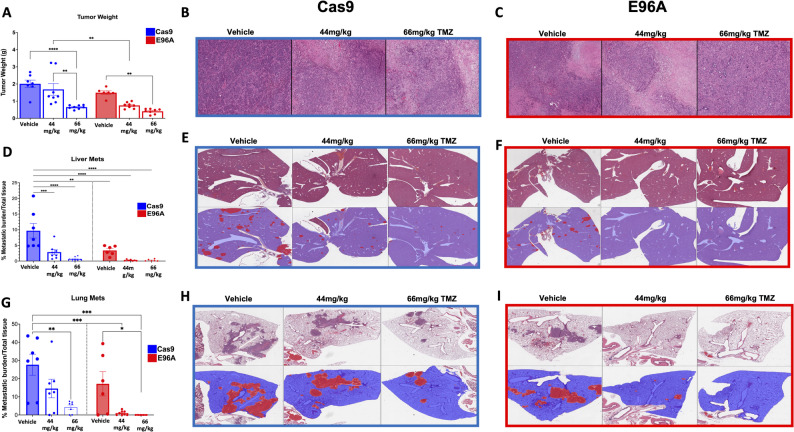
Temozolomide treatment enhances the anti-tumor and anti-metastatic effects of APE1 endonuclease deficiency in vivo. **A** Final weights of primary tumors harvested at week 5. Treatment commenced on day 7 post-orthotopic implantation and consisted of Vehicle, 44 mg/kg, or 66 mg/kg Temozolomide (TMZ) administered via oral gavage (p.o.) 5 days per week for 3 consecutive weeks. **B**, **C** Representative H&E staining of primary tumors from Cas9 control (**B**) and E96A G8 mutant (**C**) cohorts across treatment groups. **D** Quantification of hepatic metastatic burden. **E**, **F** Representative liver histology for Cas9 (**E**) and E96A (**F**) cohorts. Top rows display raw H&E staining; bottom rows display binary segmentation masks used for quantification (Red: metastatic foci; Blue: normal liver tissue). **G** Quantification of pulmonary metastatic burden. **H**, **I** Representative lung histology and segmentation masks for Cas9 (**H**) and E96A (**I**) cohorts. Data represent mean ± SEM (n³6 per group). Statistical significance was determined by one-way ANOVA with Tukey’s multiple comparisons test (*p < 0.05, **p < 0.01, ***p < 0.001, ****p < 0.0001)

While TMZ administration elicited a therapeutic response in both cohorts, the APE1^E96A^ tumors displayed a significantly greater sensitivity than controls, consistent with the in vitro susceptibility *(*Fig. 8A, D, G). Once again, at baseline (vehicle), the APE1^E96A^ tumors exhibited reduced growth and metastatic burden. However, upon treatment, this vulnerability was exacerbated. E96A mutant tumors exhibited a marked reduction in final tumor volume relative to the Cas9 group (Fig. 8A-C). Significantly, the APE1^E96A^ mice demonstrated a dose-dependent decrease in metastatic burden (Fig. 8D-I). The potency of this sensitization was underscored by the fact that the lower dose (44 mg/kg) in APE1^E96A^ mice achieved metastasis suppression equivalent to that of the maximum dose (66 mg/kg) required in wild-type Cas9 controls. Collectively, these results demonstrate that APE1 endonuclease deficiency markedly increases PDAC tumors’ sensitivity to TMZ treatment in vivo.

### Temozolomide treatment triggers temporary stress response pathways while continuously suppressing ECM and adhesion pathways in the DNA BER deficient tumors

**Fig. 9:**
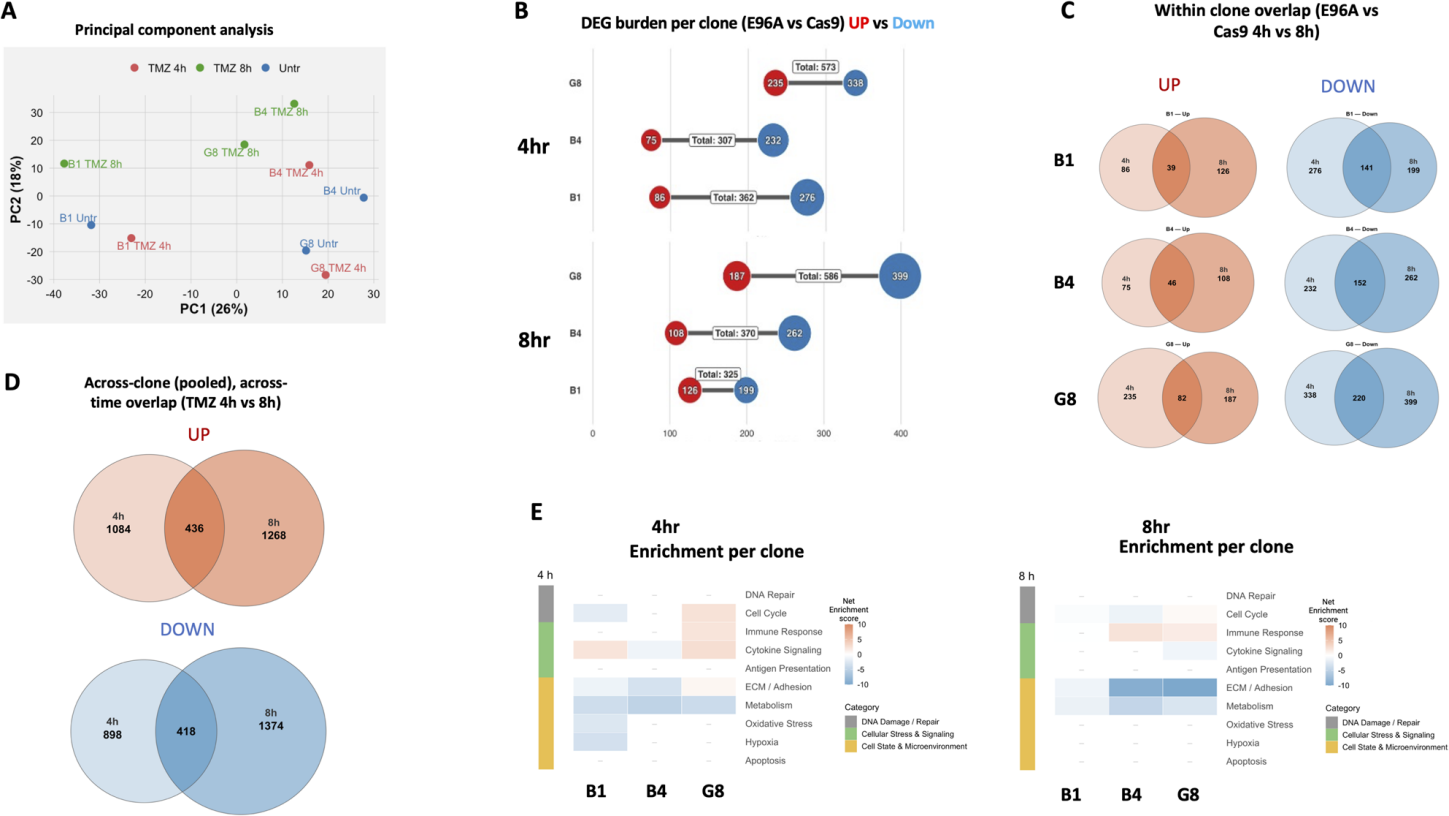
Temozolomide induces stress signaling and inhibits cell-cycle and adhesion pathways without repair compensation. **A** Principal Component Analysis (PCA) of global gene expression profiles from E96A mutant clones treated with 200µM Temozolomide (TMZ) for 4 or 8 h, compared to untreated controls. **B** Quantification of differentially expressed genes (DEGs) for each clone at 4 h and 8 h relative to untreated Cas9 controls (FDR < 0.05). **C** Venn diagrams displaying the intra-clonal intersection of DEGs identified at 4 h versus 8 h post-treatment. **D** Venn diagrams displaying the overlap of pooled DEGs (combined across all three clones) comparing the global 4 h versus 8 h transcriptional signatures. **E** Pathway enrichment analysis at 4 h (left) and 8 h (right) aggregated into ten functional biological buckets. Heatmap intensity represents the Net Enrichment Score, calculated as the difference between the -log_10_(FDR) of the top upregulated and downregulated terms (capped at ± 10). Red indicates net pathway activation; blue indicates net suppression

To define transcriptomic changes following TMZ treatment, we next analyzed the APE1^E96A^ clones collected at 4- and 8-hours post-treatment. Principal component analysis revealed a strong response to temozolomide treatment (Fig. 9A). The first principal component (PC1; 26.23% of variance) captured the dominant separation between treated and untreated samples, while PC2 (17.75%) reflected the temporal shift from 4h to 8h. Notably, B1 exhibited a clear separation from B4 and G8 (untreated and treated), consistent with previous results.

Compared to untreated Cas9, all mutant cell lines exhibited substantial transcriptional alterations, with 300–600 differentially expressed genes (DEGs; FDR < 0.05) per clone at each time point (Fig. 9B). The DEGs demonstrated more significant downregulation with TMZ treatment at both timepoints.

Within-clone comparisons showed that a large fraction of DEGs were shared between 4 h and 8 h, indicating stable temporal responses (Fig. 9C). When pooled across clones, 436 transcripts were consistently upregulated and 418 downregulated across both time points (Fig. 9D), demonstrating a conserved, time-stable response to TMZ exposure.

Pathway enrichment analysis revealed a transient stress response characterized by upregulation of cytokine signaling, particularly at 4 h. In contrast, downregulated signatures were dominated by ECM/adhesion and metabolism pathways, with the most pronounced effect at 8 h (Fig. 9E).

Overall, TMZ treatment induces a transient stress response characterized by activation of cytokine signaling and suppression of cellular proliferation and adhesion. Importantly, we observed no evidence for the transcriptional induction or protein induction of APE1-independent BER pathways in the presence or absence of TMZ treatment (Supplemental Fig. S2).

## Discussion

We generated stable human PDAC cell lines selectively deficient in APE1 endonuclease activity through homozygous knock-in of the E96A mutation. This mutation decreased incision activity by approximately 150-fold (0.6–0.8% of wild-type) yet preserved both protein expression and redox function (Fig. 1B-D), confirming that the observed phenotypes arise from BER impairment rather than changes in redox-activated transcriptional activity. Attempts to create similar cell lines with an APE1^D210A^ mutation failed to produce viable cells (Supplemental Table S1). Loss of D210 abolishes all catalytic activity, representing a stringent viability threshold for APE1 in cells consistent with the embryonic lethality observed in complete APE1 knockout mice [45]. The E96A cells likely represent the lowest viable point in a repair-deficient state, making them a useful model for examining the role of APE1 endonuclease function in living tumor cells. The survival of cells with significantly reduced, but not absent, endonuclease activity suggests that partial drug inhibition could be therapeutically feasible without the toxicity associated with total inhibition.

Assessment of the E96A cells using short-term (24-hour) cytotoxicity assays showed no increase in acute sensitivity to oxidative or alkylating agents (Fig. 2A-C), indicating that the cells are resistant to immediate cytotoxicity. Long-term evaluation, however, revealed that the E96A cell lines formed 50% fewer colonies at baseline and experienced an additional 70–90% loss following brief exposure to alkylating or oxidative stress (Fig. 2D-H). Since mammalian cells experience approximately 10,000 to 20,000 abasic sites daily, baseline reductions likely reflect the inability of the E96A cells to fully resolve endogenous lesions. The subsequent severe decline in survival following treatment reveals that further stress in this vulnerable state significantly compounds baseline reductions. This shows that while the residual APE1 endonuclease activity of the E96A cells can support basic survival, it is insufficient for sustained proliferation.

Mechanistically, this survival deficit appears to be driven by impaired genomic maintenance following the loss of endonuclease activity, rendering the E96A cells acutely stress sensitive. Following oxidative challenge, nuclear and mitochondrial DNA damage in E96A cells rose sharply, while control cells did not show a similar increase. In the Cas9 controls, resolution started immediately, with differences between genotypes observable by 2 h in nuclear DNA. Similarly, while mitochondrial lesion burden in E96A cells peaked at 12 h post-treatment, control cells had already completed repair (Fig. 3). This sustained lesion burden was accompanied by a marked reduction in cellular viability and a shift in cell death distribution toward increased membrane-compromised states, as evidenced by enrichment of the PI+/Annexin- population (Q1) (Fig. 4A-C; Supplemental Fig. S1).

Although Annexin V/PI staining does not definitively distinguish necrotic from late apoptotic processes, these findings suggest that unresolved genomic damage in E96A cells predisposes them to a loss of membrane integrity under stress, rather than progression through classical apoptotic pathways. This points towards a model in which APE1 endonuclease activity serves as a critical buffer against oxidative injury by enabling timely resolution of both nuclear and mitochondrial DNA lesions. In its absence, persistent genomic damage appears to overwhelm cellular recovery pathways, resulting in reduced viability and a shift toward membrane-compromised cell death.

Collectively, our results highlight the crucial role of APE1 endonuclease activity in maintaining genomic stability. The APE1^E96A^ cells failed to withstand brief genotoxic stress that Cas9 control cells survived, highlighting how APE1’s high catalytic capacity enables adaptive responses to environmental challenges, a plasticity vital for PDAC cell survival. This loss of adaptability was evident in the baseline transcriptomic analysis, which showed widespread suppression of pathways linked to PDAC aggressiveness (Fig. 5D). All APE1^E96A^ cell lines exhibited significant downregulation of genes involved in ECM remodeling, cell adhesion, and cell cycle progression. Notably, we saw no increase in DNA repair or other stress-response pathways, suggesting the cells are not using other repair pathways to compensate for the loss of APE1 endonuclease activity, at least at the transcriptional level. In vivo, the observed transcriptional changes translated to smaller, less invasive tumors even without treatment, alongside systemic effects: mice with E96A G8 tumors had 16.5% and 21% reductions in circulating oncogenic microRNAs miR-10b and miR-155, respectively (Fig. 6G-H). Since these miRNAs are linked to PDAC invasion and metastasis [37, 46], the concordance between decreased adhesion pathways in tumor cells and lower levels of pro-metastatic circulating miRNAs suggests APE1 endonuclease activity supports not only cell-intrinsic invasion but also tumor-host communication networks that promote metastasis.

The stress-sensitive state of the APE1^E96A^ cells persisted because APE1 endonuclease function in PDAC appears to be functionally non-redundant. Neither mRNA nor protein analyses showed induction of compensatory repair enzymes (Fig. 5 and 9, Supplemental Fig. 2), and targeted depletion of potential backup factors (APE2, PNKP) failed to further increase alkylation sensitivity in the E96A background (Supplemental Fig. 3), confirming that these enzymes do not functionally substitute for APE1. This lack of compensation likely reflects both kinetic and regulatory constraints. APE1 is among the most abundant (10⁶ to 10⁷ molecules per cell) and catalytically efficient DNA repair enzymes in mammalian cells (kcat ~ 10 s⁻¹), completing single-lesion repair within minutes [17]. Although bifunctional glycosylases can theoretically cleave abasic sites, their turnover rates are orders of magnitude slower (kcat ~ 0.001–0.01 s⁻¹), rendering them kinetically inadequate to compensate for APE1 loss [47–49].

The therapeutic vulnerability of E96A cells is defined by the nature of the genotoxic insult. We observed a clear sensitivity hierarchy- TMZ > oxaliplatin ≈ cisplatin > carboplatin- that correlates directly with each agent’s ability to generate abasic sites (Fig. 7). Platinum-based therapies primarily kill tumor cells through DNA crosslinking; however, they vary in their secondary production of ROS and oxidative damage. Carboplatin generates little detectable ROS and oxidative base damage and is minimally affected by BER impairment, whereas oxaliplatin and cisplatin produce substantial ROS and oxidative lesions, leading to increased toxicity when BER is compromised [50]. TMZ, which causes methyl adducts that form abasic sites needing APE1-mediated incision for repair, proved most potent. Capitalizing on this sensitivity, we demonstrated that TMZ treatment decreased E96A tumor mass by 78% in vivo, compared to 45% in controls (*p* < 0.001, Fig. 8B). The 66 mg/kg dose used corresponds to the standard human clinical regimen of 150–200 mg/m² [42–44], indicating that therapeutic enhancement is achievable at approved doses without toxic escalation. Transcriptomic analysis of E96A cells after TMZ treatment revealed a transient activation of stress response pathways, characterized by elevated cytokine signaling, along with sustained suppression of ECM/adhesion and metabolic programs (Fig. 9E). Again, no compensatory repair pathways were activated. These findings show that inhibiting APE1 endonuclease activity can deliver significant therapeutic benefit at doses feasible in clinical settings, potentially enabling dose-sparing strategies that minimize systemic toxicity while maintaining efficacy.

### APE1 expression in human PDAC and clinical relevance

The therapeutic potential of targeting APE1 is supported by clinical data showing elevated APE1 expression in human PDAC tissues compared to normal pancreas [51, 52]. Additionally, APE1 overexpression has been linked to chemoresistance in several cancer types [19, 53], indicating that high APE1 levels could serve as both a prognostic and predictive marker for APE1-targeted treatments. Our finding that cells lacking endonuclease activity are more sensitive to chemotherapy drugs suggests that inhibiting APE1 might help re-sensitize chemoresistant PDAC tumors, supporting further research using patient-derived models from treatment-resistant cases.

Approximately one-quarter of PDAC tumors have homologous recombination deficiencies (HRD) caused by BRCA1/2, PALB2, or ATM mutations [4, 54]. In this context, combining BER inhibition with DNA-damaging agents may create synergistic vulnerabilities analogous to the clinical success of PARP inhibitors in BRCA-mutant cancers [55]. Baseline APE1 expression levels, which vary widely across PDAC tumors, might also predict response, with tumors that have high expression potentially being more dependent.

Despite this strong biological rationale, clinical translation of APE1-targeted therapies remains limited. APE1 inhibitor development has focused on its two distinct functional domains. The redox inhibitor APX3330 (E3330), which disrupts APE1’s redox regulation of transcription factors including NF-κB, HIF-1α, and AP-1, has advanced into early-phase clinical trials and demonstrated favorable tolerability, although clinical efficacy has thus far been modest [56]. Ongoing efforts are focused on improving potency through next-generation compounds [22]. Importantly, APX3330 does not inhibit APE1 endonuclease activity, leaving the DNA repair function of APE1 therapeutically unaddressed.

In contrast, efforts to target the endonuclease domain have largely remained preclinical. Small-molecule inhibitors such as CRT0044876 and related compounds have demonstrated proof-of-concept activity in vitro [57], while methoxyamine (TRC102), which indirectly targets the base excision repair pathway by modifying abasic sites, has entered clinical trials in combination with alkylating agents [58, 59] with limited clinical efficacy. However, direct endonuclease inhibitors have faced significant challenges, including limited potency, suboptimal pharmacokinetics, and difficulty achieving selective inhibition of APE1 without off-target toxicity [24, 60].

Our findings provide genetic evidence that selective disruption of APE1 endonuclease activity is sufficient to sensitize PDAC cells to clinically relevant DNA-damaging agents. These results confirm APE1 endonuclease function as a biologically validated yet pharmacologically underexploited therapeutic target and support continued efforts to develop more potent, selective, and clinically viable inhibitors of this domain.

### Study limitations and future directions

Although the E96A cell lines are useful for studying APE1 endonuclease activity, they may not fully capture the effects of immediate pharmacologic inhibition. This study was conducted in a single PDAC cell line (Pa03C) and therefore does not encompass the full molecular heterogeneity of pancreatic cancer. While this model enabled precise genetic interrogation of APE1 endonuclease function, extending these findings to additional PDAC models will be an important future direction. Additionally, long-term adaptation in engineered clones could conceal stress responses that would occur in drug-treated tumors. For example, the variation observed among E96A clones suggests that compensatory mutations or epigenetic changes can partially offset BER deficiency over time. Short-term drug treatment may uncover vulnerabilities that are not visible in adapted cell lines. Comparative studies using pharmacological APE1 inhibitors in parental PDAC cells will be needed to confirm that genetic and drug-based inhibition yield similar outcomes. While in vivo studies in this work focused on temozolomide, extending these findings to additional clinically relevant agents, such as platinum-based therapies, will be important to further establish the therapeutic scope of APE1 endonuclease inhibition.

We observed biological heterogeneity among the cell lines. Despite harboring identical APE1^E96A^ mutations, the three derived clones exhibited notable phenotypic variation. Clone B1 showed significantly fewer differentially expressed genes (263 DEGs) compared to B4 (671 DEGs) and G8 (869 DEGs) (Fig. 5B) and displayed an intermediate phenotype with weaker metastatic suppression (Fig. 6C-F). This heterogeneity, a common feature of clonal selection, likely reflects clone-specific adaptations or secondary genomic changes that partially buffer BER deficiency. Crucially, however, all three APE1^E96A^ clones demonstrated significant sensitivity to genotoxic stress and showed similar reductions in tumor size, confirming that the core phenotype is mutation-driven rather than clone-specific.

An additional limitation was the use of immunodeficient NSG mice, which prevents evaluation of immune-mediated effects of APE1 inhibition. Furthermore, both in vitro and in vivo models used in this study lack key components of the pancreatic tumor microenvironment, particularly cancer-associated fibroblasts (CAFs) and the dense fibrotic stroma characteristic of PDAC. These stromal interactions can influence tumor cell transcriptional programs and responses to genotoxic stress. Future studies using immunocompetent and stromal-rich models will be important to determine how microenvironmental factors modulate APE1 dependency and therapeutic response.

## Conclusions

This study provides the first direct in vivo evidence that selectively impairing APE1 endonuclease activity, independent of its redox function, significantly reduces PDAC tumor growth, metastatic potential, and enhances chemotherapeutic efficacy. Our results show that impairing APE1 endonuclease activity compromises both mitochondrial and nuclear genome integrity and induces systemic changes in tumors, emphasizing APE1’s crucial role in PDAC biology and aggressiveness. These findings identify APE1 endonuclease activity as a distinct and targetable vulnerability in PDAC. The observed dose-sparing effect with the clinical agent TMZ, along with the lack of compensatory adaptation, suggests that APE1 inhibitors could both enhance existing chemotherapy and expand treatment options for patients with resistant disease. The absence of activated repair pathways and the suppression of ECM and adhesion programs further indicate that targeting APE1 may improve therapeutic efficacy and reduce metastatic spread. Developing and clinically testing selective APE1 endonuclease inhibitors offers a promising approach to targeting a fundamental weakness in PDAC biology.

## Supplementary Information

Supplementary Material 1.Supplemental Fig. 1: Loss of APE1 endonuclease makes cells more sensitive to necrosis caused by alkylating agents. (A) Representative flow cytometry plots of Annexin V/Propidium Iodide (PI) staining in Cas9 control and E96A cells following exposure to 900µM Methyl methanesulfonate (MMS). Cells were analyzed at 24 h and 48 h post-treatment. (B-C) Quantification of cell fate distributions at (B) 24 h and (C) 48 h. Stacked bars display the proportion of viable (Q4), early apoptotic (Q3), late apoptotic (Q2), and necrotic (Q1) cells. Data represent mean ± SEM (n = 3). Statistical significance was determined by two-way ANOVA with Tukey’s multiple comparisons. Asterisks indicate significant differences in cell viability (Q4) between genotypes (**p < 0.01). Hashes (#) indicate a significant increase in necrosis (Q1) relative to the untreated Cas9 control (#*p* < 0.05, ###*p* < 0.001).

Supplementary Material 2. Supplemental Fig. 2: The expression of BER complex proteins remains unchanged in E96A mutants. (Left) Representative Western blot analysis of key Base Excision Repair (BER) and Single-Strand Break Repair (SSBR) proteins (PNKP, TDP1, DNA Ligase III, XRCC1, and PARP1) in Cas9 control and E96A mutant cell lines. (Right) Densitometric quantification of protein abundance normalized to Vinculin and expressed relative to the Cas9 control. Data represent mean ± SEM (n = 3). Statistical significance was determined by one-way ANOVA (ns, not significant).

Supplementary Material 3. Supplemental Fig. 3: Knockdown of potential backup enzymes does not restore genotoxic sensitivity in E96A cells. (A) Effect of APEX2 knockdown. (Top) Representative images of methylene blue-stained colonies and corresponding binary segmentation masks for Cas9 control and E96A (clone B4) cells transfected with Scramble (SCR) or APEX2-targeting siRNA, followed by exposure to MMS (225µM, 30 min pulse). (Bottom Left) Validation of APEX2 knockdown by qPCR, showing relative APEX2 mRNA expression (ΔΔCt) following transfection with SCR or siAPEX2 in Cas9 and E96A (clone B4) cell lines (mean ± SEM). (Bottom Right) Colony area quantification (normalized to untreated SCR control for each cell line) is shown (mean ± SEM; n = 3). (B) Effect of PNKP knockdown. (Top) Representative images and segmentation masks for SCR and siPNKP transfected cells. (Bottom Left) Western blot validation of PNKP knockdown (55 kDa) with densitometric quantification. B4 and Cas9 cell lysates transfected with SCR or siPNKP were probed for PNKP and Vinculin (120 kDa; loading control). Data represent mean ± SEM; n = 3. (Bottom Right) Quantification of colony area. Data represent mean ± SEM; *n* = 3. No significant differences were observed between SCR and target-specific knockdown conditions within treatment groups (one-way ANOVA; *p* > 0.05).

Supplementary Material 4. Supplemental Table S1: Generation and Viability Outcomes of APE1 Knock-In Mutant PDAC Cell Lines.

Supplementary Material 5. Supplemental Table S2: Guide RNA and donor sequences used for E96A CRISPR knock-in.

Supplementary Material 6. Supplemental Table S3: List of antibodies utilized.

Supplementary Material 7. Supplemental Table S4: List of primers and siRNA utilized.

## Data Availability

The RNA-seq datasets generated and/or analyzed during the current study are available in the NCBI Gene Expression Omnibus (GEO) repository, Accession Number GSE311737 and GSE311738. https://www.ncbi.nlm.nih.gov/geo/query/acc.cgi? acc=GSE311737 and https://www.ncbi.nlm.nih.gov/geo/query/acc.cgi? acc=GSE311738. The source data underlying the tumor growth and weight measurements (Figs. 6 and 8) are provided in Additional File 1. The remaining datasets used and/or analyzed during the current study are available from the corresponding author on reasonable request.

## Abbreviations

APE1: Apurinic/Apyrimidinic Endonuclease 1
APE2: Apurinic/Apyrimidinic Endonuclease 2
AP: Abasic/Apurinic/Apyrimidinic (sites)
ATM: Ataxia Telangiectasia Mutated
Au TNPs: Gold Triangular Nanoprisms
BER: Base Excision Repair
BRCA1/2: Breast Cancer gene 1/2
C65A: Cysteine 65 to Alanine mutation
C3B: Collaborative Core for Cancer Bioinformatics
Cas9: CRISPR-associated protein 9
cDNA: Complementary DNA
COL4A1: Collagen Type IV Alpha 1 Chain
COL4A2: Collagen Type IV Alpha 2 Chain
CRISPR: Clustered Regularly Interspaced Short Palindromic Repeats
D210A: Aspartic Acid 210 to Alanine mutation
DDR: DNA Damage Response
DEGs: Differentially Expressed Genes
E96A: Glutamic Acid 96 to Alanine mutation
ECM: Extracellular Matrix
FBS: Fetal Bovine Serum
FDR: False Discovery Rate
FITC: Fluorescein Isothiocyanate
FOLFIRINOX: FOLinic acid, Fluorouracil, IRINotecan, OXaliplatin (chemotherapy regimen)
GENCODE: Encyclopedia of DNA Elements gene annotation
GEO: Gene Expression Omnibus
GSEA: Gene Set Enrichment Analysis
H&E: Hematoxylin and Eosin
HIF-1α: Hypoxia-Inducible Factor 1-alpha
HR: Homologous Recombination
HRP: Horseradish Peroxidase
HT: Hexanethiol
IACUC: Institutional Animal Care and Use Committee
ICE: Inference of CRISPR Edits
KEGG: Kyoto Encyclopedia of Genes and Genomes
KPC: KRAS, p53, Cre (mouse model)
KRAS: Kirsten Rat Sarcoma viral oncogene homolog
LSPR: Localized Surface Plasmon Resonance
MC: Merocyanine
miR-10b: microRNA-10b
miR-155: microRNA-155
NFκB: Nuclear Factor kappa B
MMS: Methyl methanesulfonate
MSigDB: Molecular Signatures Database
miRNA: microRNA
mtDNA: Mitochondrial DNA
PALB2: Partner and Localizer of BRCA2
PARP 1: Poly (ADP-ribose) Polymerase 1
PBS: Phosphate-Buffered Saline
PCA: Principal Component Analysis
PDAC: Pancreatic Ductal Adenocarcinoma
PDB: Protein Data Bank
PD-1: Programmed cell Death protein 1
PD-L1: Programmed Death-Ligand 1
PI: Propidium Iodide
PNKP: Polynucleotide Kinase 3’-Phosphatase
PVDF: Polyvinylidene Fluoride
qRT-PCR: Quantitative Real-Time Polymerase Chain Reaction
Ref-1: Redox Effector Factor-1
RNPs: Ribonucleoprotein Complexes
ROS: Reactive Oxygen Species
SDS-PAGE: Sodium Dodecyl Sulfate Polyacrylamide Gel Electrophoresis
SEM: Standard Error of the Mean
sgRNA: Single-Guide RNA
siRNA: Small Interfering RNA
SP: Spiropyran
SP-HT: Spiropyran-Hexanethiol
SSBR: Single-Strand Break Repair
ssODN: Single-Stranded Oligodeoxynucleotide
STAR: Spliced Transcripts Alignment to a Reference
STR: Short Tandem Repeat
TBST: Tris-Buffered Saline with Tween-20
TDP1: Tyrosyl-DNA Phosphodiesterase 1
TF: Transcription Factor
TME: Tumor Microenvironment
TMM: Trimmed Mean of M-values
TMZ: Temozolomide
TNFα: Tumor Necrosis Factor alpha
UV: Ultraviolet
XRCC1: X-ray Repair Cross-Complementing protein 1
