# Supplementary figures and images for: Targeting APE1 endonuclease activity impairs metastasis and enhances genotoxic therapy response in pancreatic cancer
Source: J Exp Clin Cancer Res. 2026 Apr 2;45:123. doi: 10.1186/s13046-026-03705-7 (PMC13188706; doi:10.1186/s13046-026-03705-7)

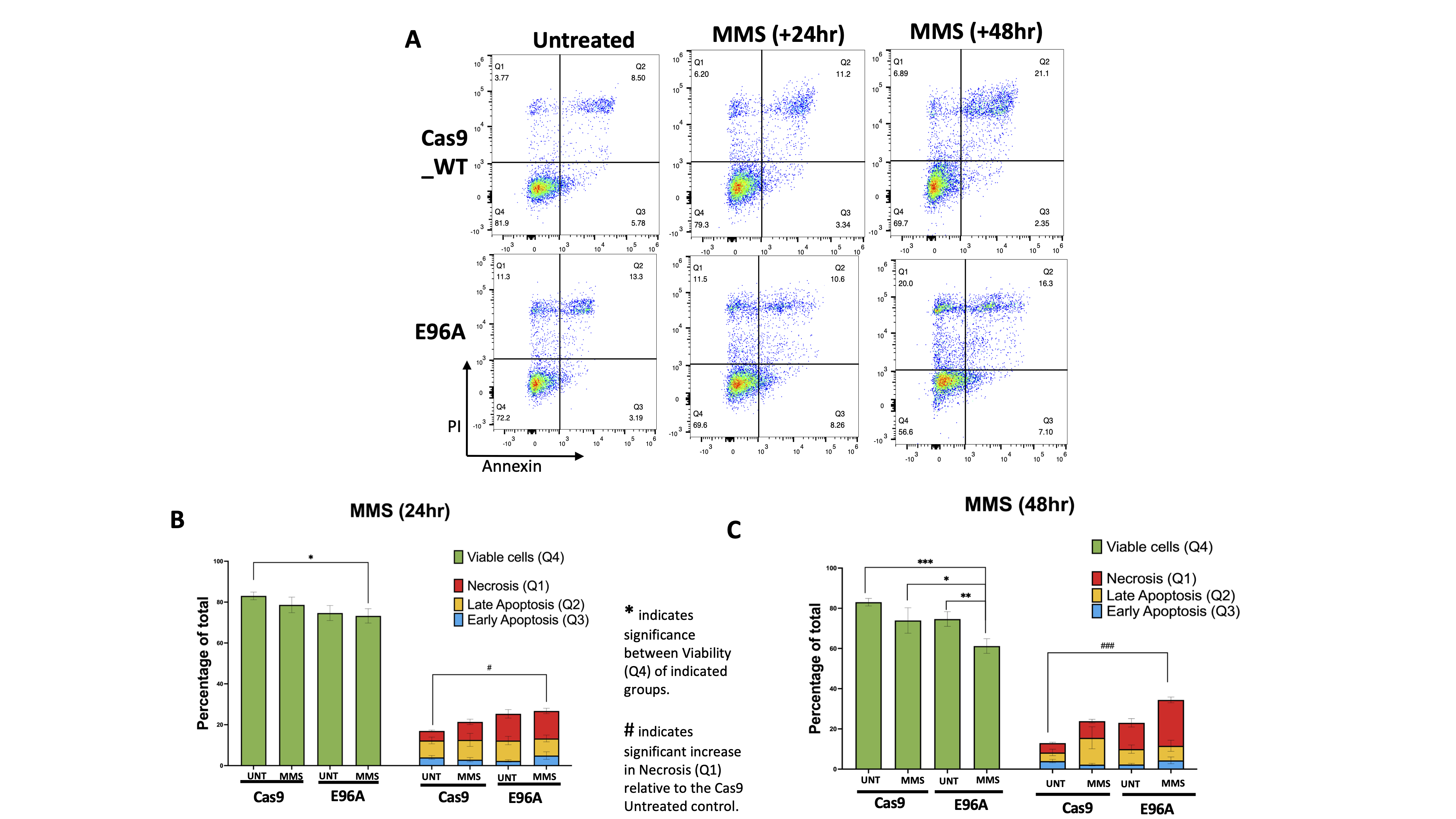

Supplement: Supplementary file 1 — Supplementary Material 1.Supplemental Fig. 1: Loss of APE1 endonuclease makes cells more sensitive to necrosis caused by alkylating agents. (A) Representative flow cytometry plots of Annexin V/Propidium Iodide (PI) staining in Cas9 control and E96A cells following exposure to 900µM Methyl methanesulfonate (MMS). Cells were analyzed at 24 h and 48 h post-treatment. (B-C) Quantification of cell fate distributions at (B) 24 h and (C) 48 h. Stacked bars display the proportion of viable (Q4), early apoptotic (Q3), late apoptotic (Q2), and necrotic (Q1) cells. Data represent mean ± SEM (n = 3). Statistical significance was determined by two-way ANOVA with Tukey’s multiple comparisons. Asterisks indicate significant differences in cell viability (Q4) between genotypes (**p < 0.01). Hashes (#) indicate a significant increase in necrosis (Q1) relative to the untreated Cas9 control (#p < 0.05, ###p < 0.001). [file 13046_2026_3705_MOESM1_ESM.tiff]

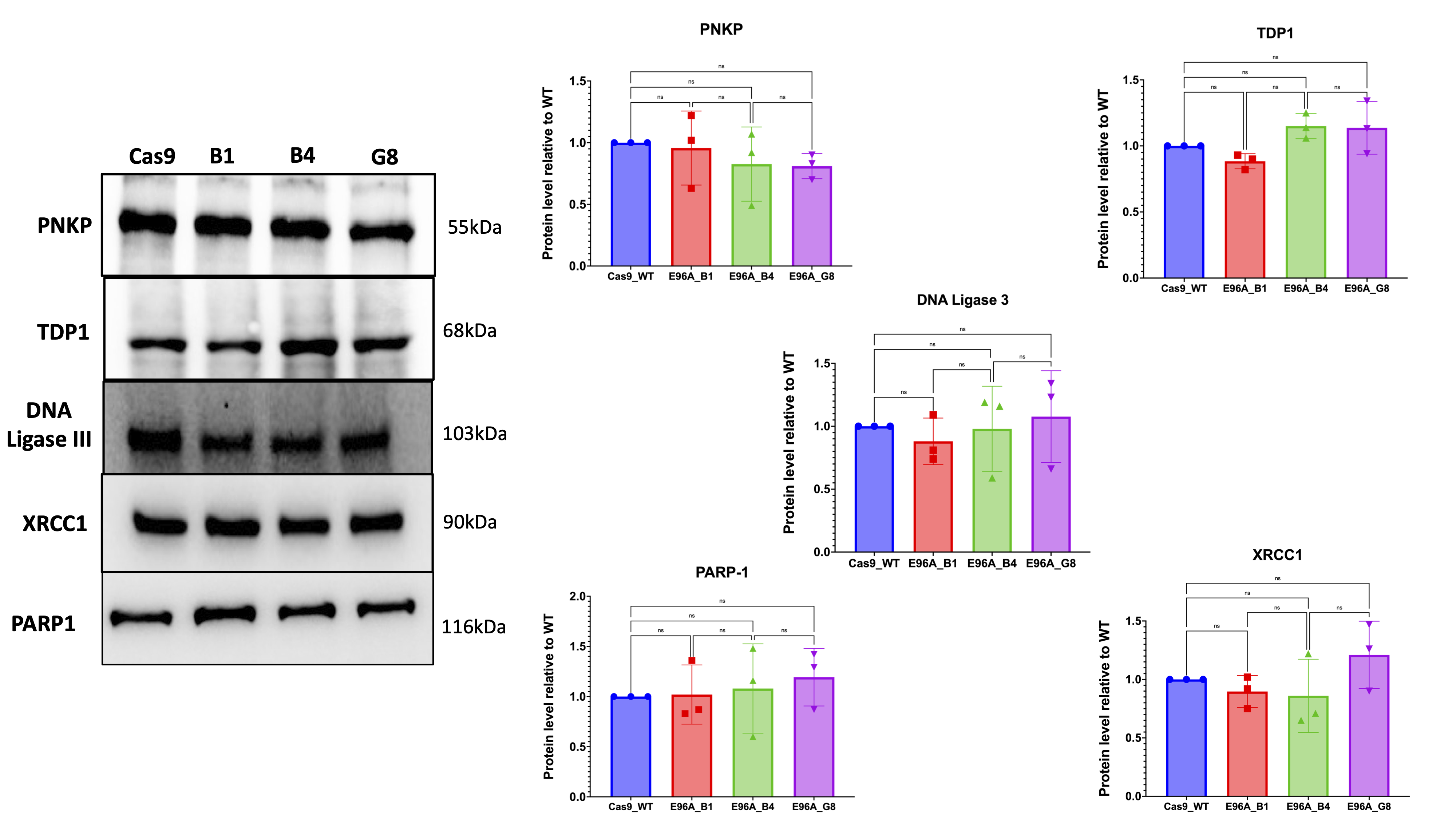

Supplement: Supplementary file 2 — Supplementary Material 2. Supplemental Fig. 2: The expression of BER complex proteins remains unchanged in E96A mutants. (Left) Representative Western blot analysis of key Base Excision Repair (BER) and Single-Strand Break Repair (SSBR) proteins (PNKP, TDP1, DNA Ligase III, XRCC1, and PARP1) in Cas9 control and E96A mutant cell lines. (Right) Densitometric quantification of protein abundance normalized to Vinculin and expressed relative to the Cas9 control. Data represent mean ± SEM (n = 3). Statistical significance was determined by one-way ANOVA (ns, not significant). [file 13046_2026_3705_MOESM2_ESM.tiff]

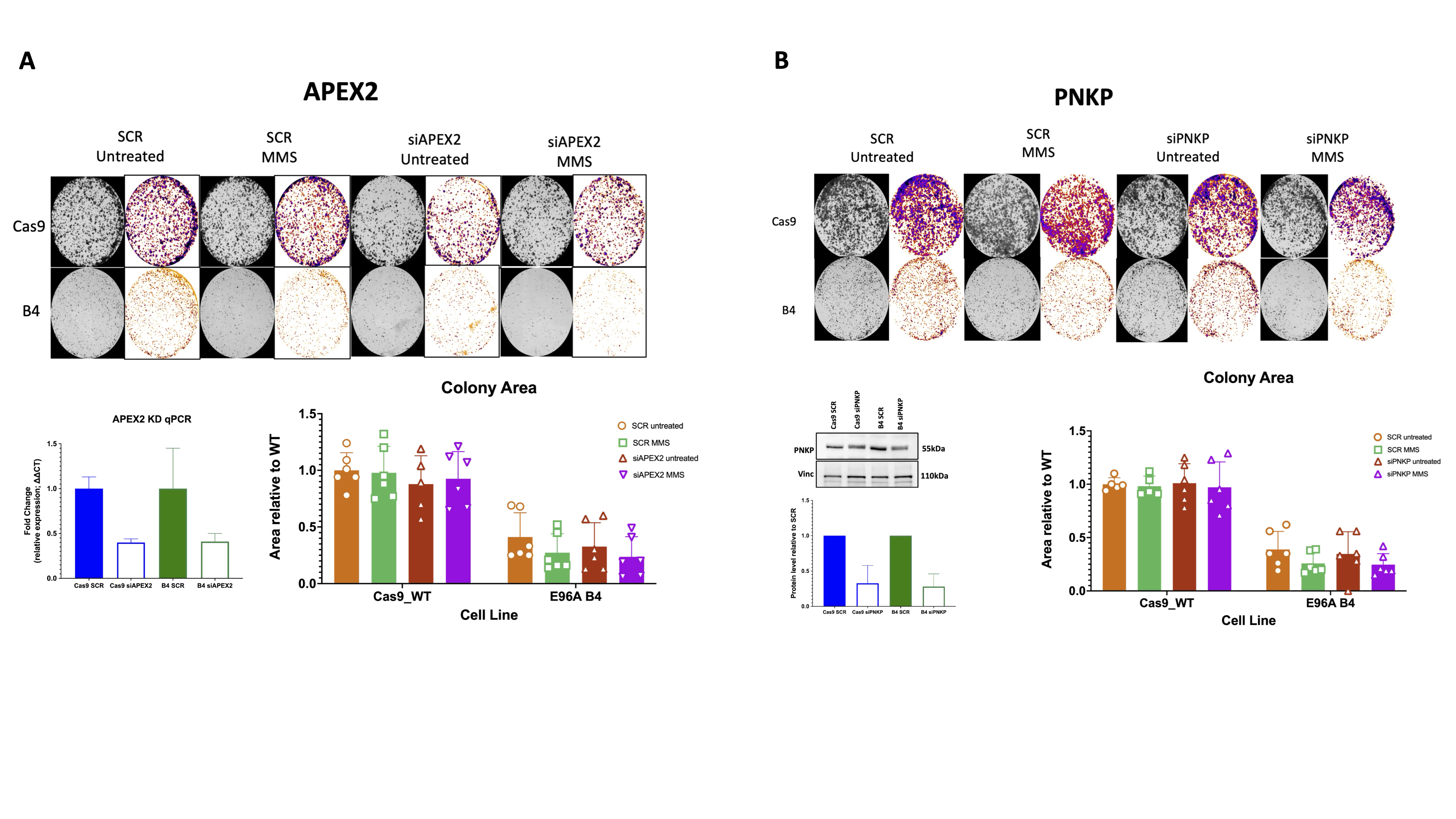

Supplement: Supplementary file 3 — Supplementary Material 3. Supplemental Fig. 3: Knockdown of potential backup enzymes does not restore genotoxic sensitivity in E96A cells. (A) Effect of APEX2 knockdown. (Top) Representative images of methylene blue-stained colonies and corresponding binary segmentation masks for Cas9 control and E96A (clone B4) cells transfected with Scramble (SCR) or APEX2-targeting siRNA, followed by exposure to MMS (225µM, 30 min pulse). (Bottom Left) Validation of APEX2 knockdown by qPCR, showing relative APEX2 mRNA expression (ΔΔCt) following transfection with SCR or siAPEX2 in Cas9 and E96A (clone B4) cell lines (mean ± SEM). (Bottom Right) Colony area quantification (normalized to untreated SCR control for each cell line) is shown (mean ± SEM; n = 3). (B) Effect of PNKP knockdown. (Top) Representative images and segmentation masks for SCR and siPNKP transfected cells. (Bottom Left) Western blot validation of PNKP knockdown (55 kDa) with densitometric quantification. B4 and Cas9 cell lysates transfected with SCR or siPNKP were probed for PNKP and Vinculin (120 kDa; loading control). Data represent mean ± SEM; n = 3. (Bottom Right) Quantification of colony area. Data represent mean ± SEM; n = 3. No significant differences were observed between SCR and target-specific knockdown conditions within treatment groups (one-way ANOVA; p > 0.05). [file 13046_2026_3705_MOESM3_ESM.tiff]

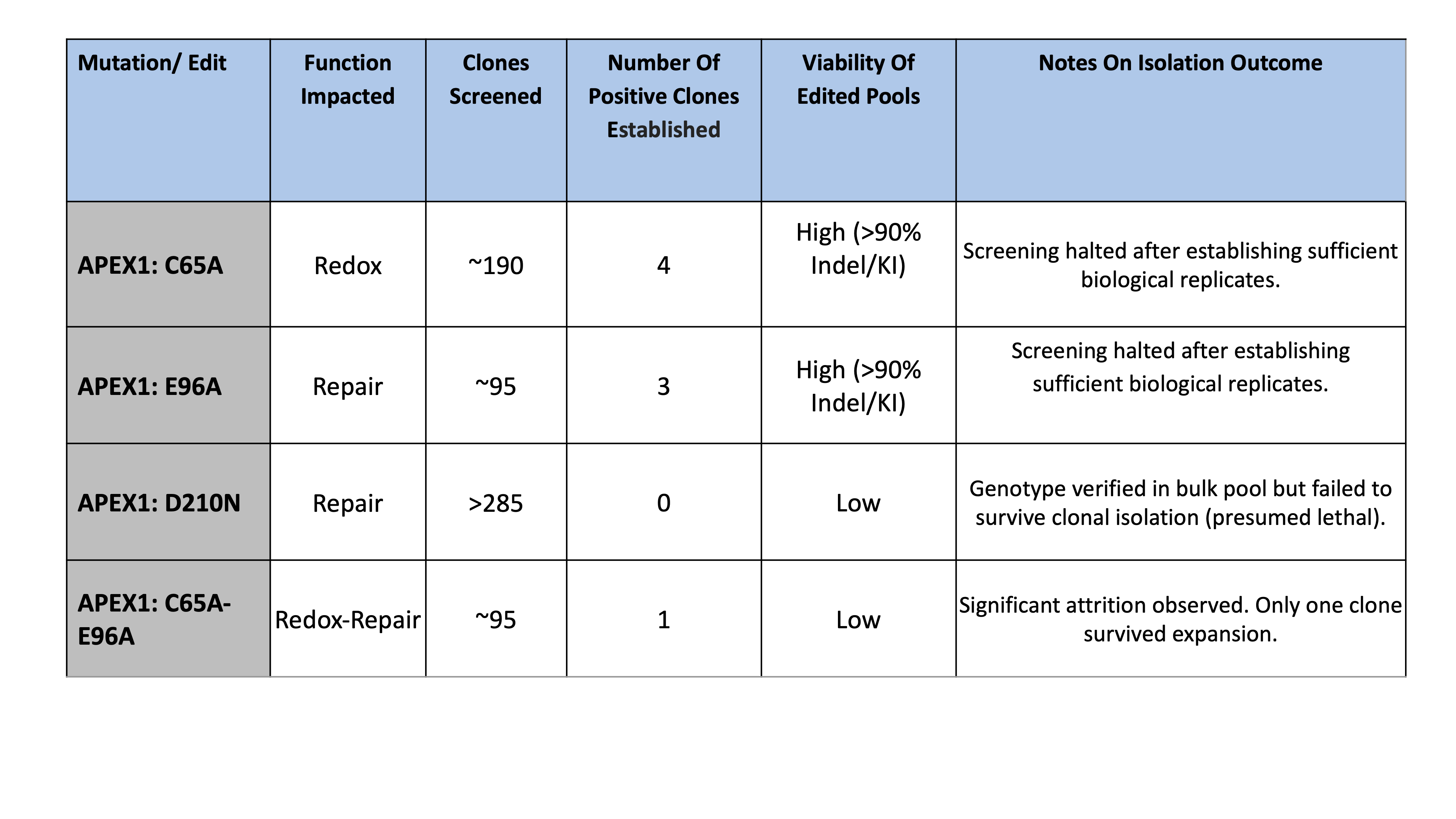

Supplement: Supplementary file 4 — Supplementary Material 4. Supplemental Table S1: Generation and Viability Outcomes of APE1 Knock-In Mutant PDAC Cell Lines. [file 13046_2026_3705_MOESM4_ESM.tiff]

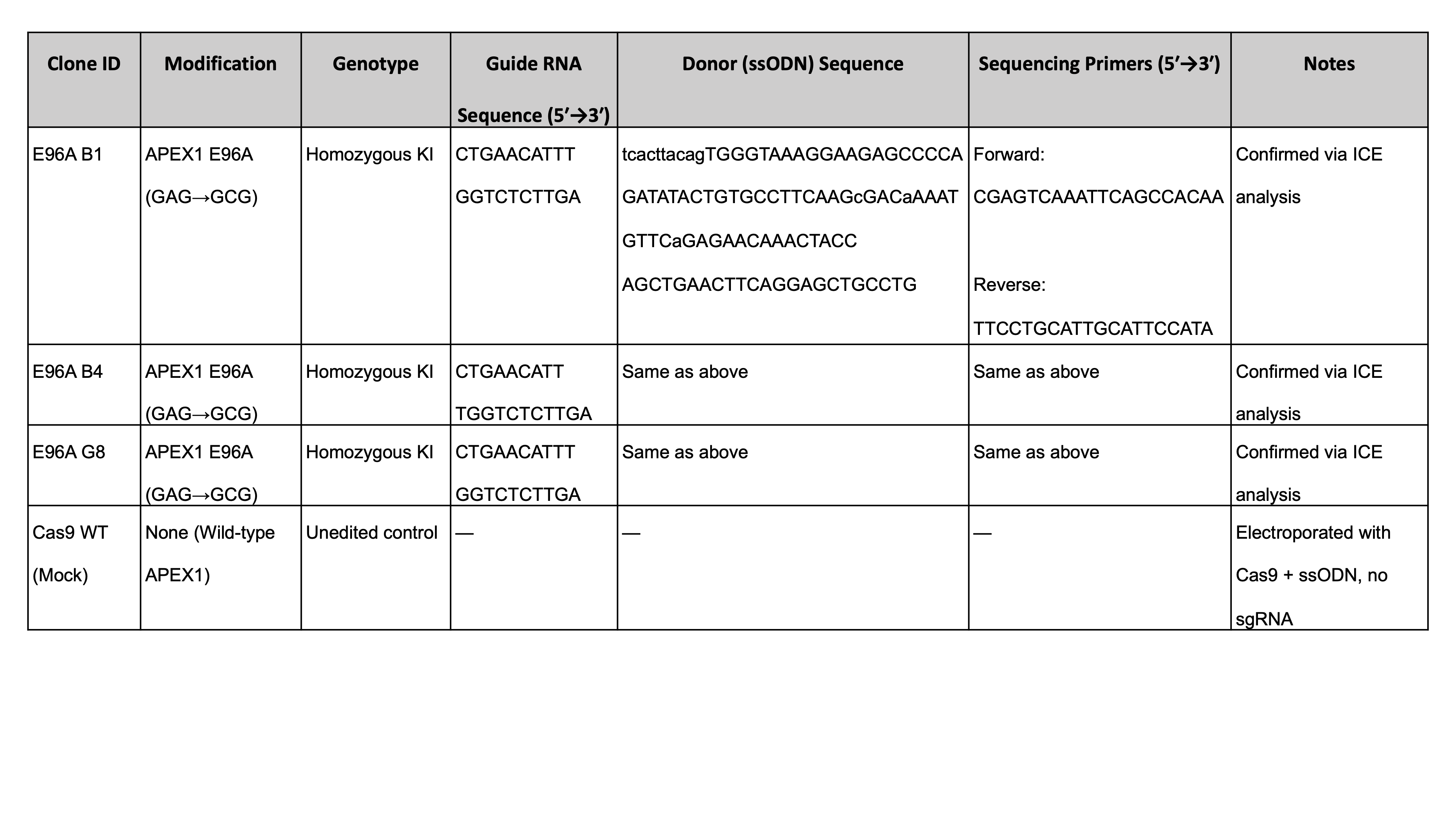

Supplement: Supplementary file 5 — Supplementary Material 5. Supplemental Table S2: Guide RNA and donor sequences used for E96A CRISPR knock-in. [file 13046_2026_3705_MOESM5_ESM.tiff]

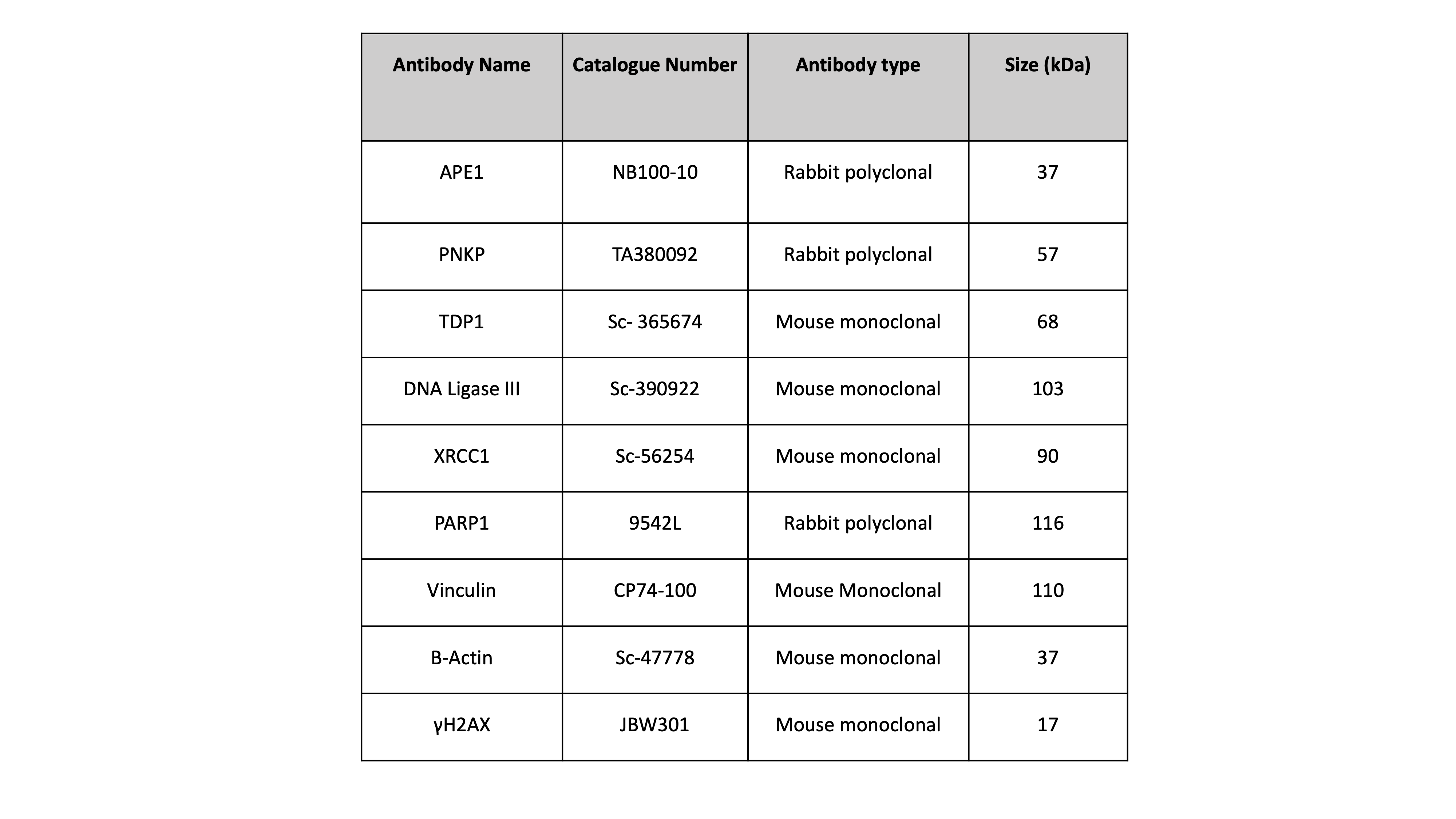

Supplement: Supplementary file 6 — Supplementary Material 6. Supplemental Table S3: List of antibodies utilized. [file 13046_2026_3705_MOESM6_ESM.tiff]

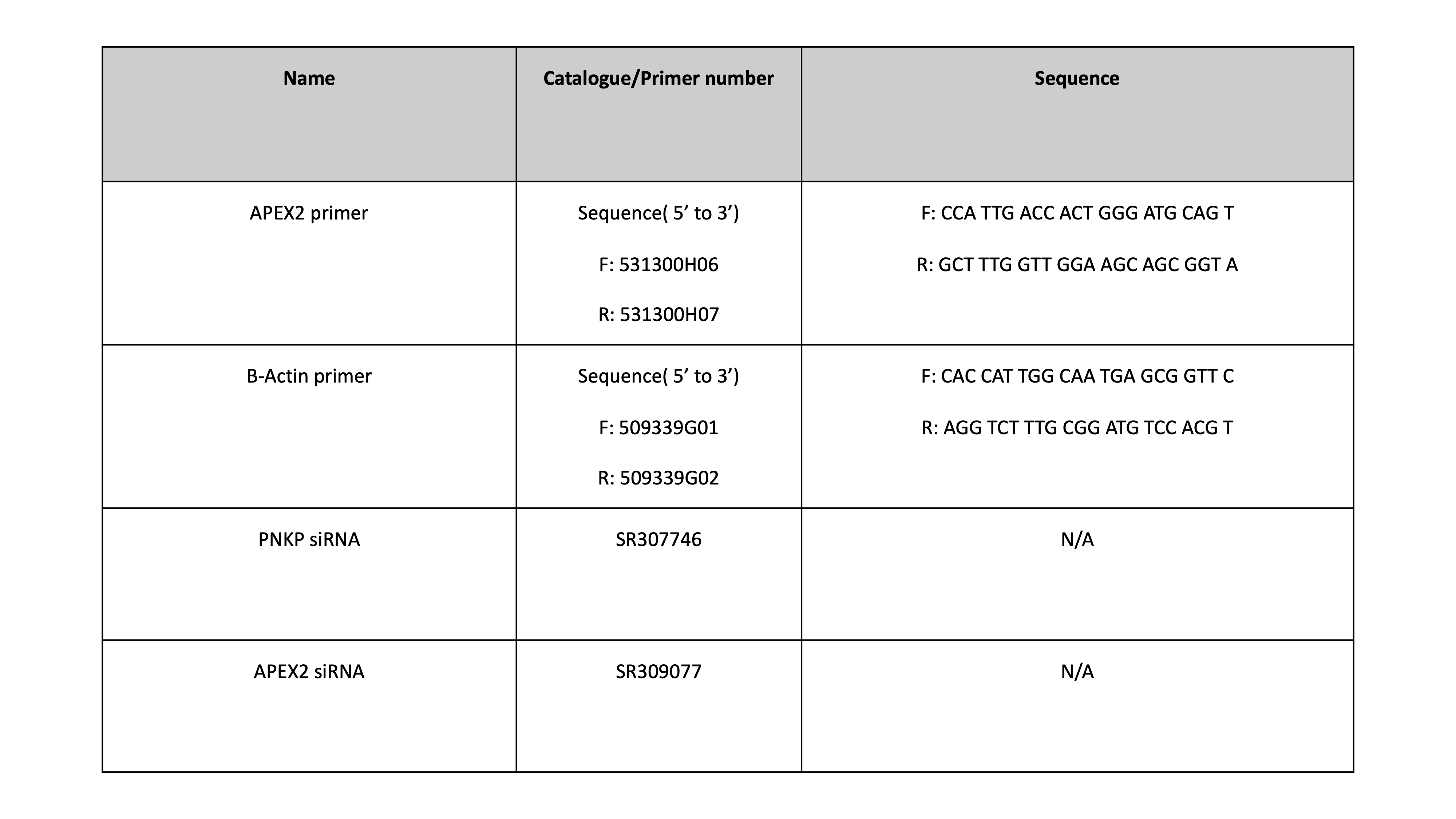

Supplement: Supplementary file 7 — Supplementary Material 7. Supplemental Table S4: List of primers and siRNA utilized. [file 13046_2026_3705_MOESM7_ESM.tiff]
